# Characterization of T-Circles and Their Formation Reveal Similarities to *Agrobacterium* T-DNA Integration Patterns

**DOI:** 10.3389/fpls.2022.849930

**Published:** 2022-05-06

**Authors:** Kamy Singer, Lan-Ying Lee, Jing Yuan, Stanton B. Gelvin

**Affiliations:** Department of Biological Sciences, Purdue University, West Lafayette, IN, United States

**Keywords:** *Agrobacterium*, *Arabidopsis thaliana*, Ku80, *Nicotiana benthamiana*, T-circles, T-DNA integration, VirD2

## Abstract

*Agrobacterium* transfers T-DNA to plants where it may integrate into the genome. Non-homologous end-joining (NHEJ) has been invoked as the mechanism of T-DNA integration, but the role of various NHEJ proteins remains controversial. Genetic evidence for the role of NHEJ in T-DNA integration has yielded conflicting results. We propose to investigate the formation of T-circles as a proxy for understanding T-DNA integration. T-circles are circular double-strand T-DNA molecules, joined at their left (LB) and right (RB) border regions, formed in plants. We characterized LB-RB junction regions from hundreds of T-circles formed in *Nicotiana benthamiana* or *Arabidopsis thaliana*. These junctions resembled T-DNA/plant DNA junctions found in integrated T-DNA: Among complex T-circles composed of multiple T-DNA molecules, RB-RB/LB-LB junctions predominated over RB-LB junctions; deletions at the LB were more frequent and extensive than those at the RB; microhomology was frequently used at junction sites; and filler DNA, from the plant genome or various *Agrobacterium* replicons, was often present between the borders. Ku80 was not required for efficient T-circle formation, and a VirD2 *ω* mutation affected T-circle formation and T-DNA integration similarly. We suggest that investigating the formation of T-circles may serve as a surrogate for understanding T-DNA integration.

## Introduction

*Agrobacterium tumefaciens* is known for its ability to genetically transform plants. During transformation, *Agrobacterium* transfers a segment of DNA [T (transferred)-DNA] into plant cells where T-DNA may integrate into the plant genome. T-DNA resides on the *Agrobacterium* tumor inducing (Ti) or rhizogenic (Ri) plasmid which also contains virulence (*vir*) genes important for transformation. The T-DNA region of Ti/Ri is delimited by two 25 base pair (bp) border repeats, the right and left borders (RB and LB). Natural T-DNAs harbor genes that induce tumors and specify the production of opines, but do not contain genes required for transformation. In modified laboratory strains, T-DNA may be cloned into a binary vector and co-reside with a separate plasmid containing *vir* genes (Gelvin, 2010, 2017, 2021; Nester, 2015; Singer, 2018; Lacroix and Citovsky, 2019).

To initiate T-DNA transfer, VirD2 protein nicks the T-DNA border regions between nucleotides 3 and 4, releasing T-DNA as a single-strand molecule (T-strand) from the Ti/Ri or binary plasmid (Wang et al., 1987). VirD2 remains covalently attached to the 5′ end, the RB side, of the released T-strand (Ward and Barnes, 1988; Young and Nester, 1988; Durrenberger et al., 1989; Howard et al., 1989). VirD2 leads the T-strand through a type IV secretion system into the plant cell and the nucleus (Cascales and Christie, 2004; van Kregten et al., 2009). It is thought that after a T-strand enters the plant cytoplasm it is coated by VirE2 to form a T-complex. This proposed complex protects T-DNA and facilitates its trafficking to the nucleus (Howard and Citovsky, 1990; Yusibov et al., 1994; Rossi et al., 1996).

How T-DNA integrates into the plant genome is a major unanswered question of *Agrobacterium*-mediated transformation. One model suggests that T-strands are first converted into double-strand molecules that subsequently integrate. Other models suggest that T-strands invade plant DNA at a nick or double-strand break site, search for microhomology, then use plant proteins to replicate and ligate T-DNA into the genome using plant DNA as a primer (Mayerhofer et al., 1991; Tinland and Hohn, 1995; Tinland, 1996; Tzfira et al., 2004; van Kregten et al., 2016; Gelvin, 2017, 2021). All these models posit that T-DNA integrates into plant genomic nicks or double-strand breaks.

It is likely that integration of T-DNA into plant chromosomes is mediated by host factors. However, the identity of these proteins and their mechanistic roles have yet to be elucidated. On this account, the literature is controversial. Several groups proposed that Ku80 or DNA ligase IV, key components of the classical NHEJ DNA repair pathway, are important for T-DNA integration (Friesner and Britt, 2003; Li et al., 2005; Jia et al., 2012; Mestiri et al., 2014; Saika et al., 2014). Other studies showed no decrease in stable transformation frequency using *Arabidopsis ku80* and other NHEJ mutants (Gallego et al., 2003; van Attikum et al., 2003), and two studies noted an increase in stable transformation using NHEJ mutants and Virus Induced Gene Silencing (VIGS) lines (Vaghchhipawala et al., 2012; Park et al., 2015). Similarly, one study indicated an essential role for DNA polymerase θ (PolQ) in T-DNA integration (van Kregten et al., 2016), whereas another study showed that *Arabidopsis* and rice *polQ* mutants could be stably transformed, and that the amount of integrated T-DNA was 50–90% of that seen in wild-type plants (Nishizawa-Yokoi et al., 2021).

VirD2 may play a role in T-DNA integration (Tinland et al., 1995). Alteration of four amino acids near the VirD2 C-terminus, termed the omega (ω) domain, to serines almost completely eliminated T-DNA integration while reducing transient transformation only 4-to-5 fold (Shurvinton et al., 1992; Narasimhulu et al., 1996; Mysore et al., 1998). However, the role of the *ω* domain in T-DNA integration is not clear (Bravo-Angel et al., 1998).

Double-strand circular T-DNA molecules (T-circles) have been isolated from *Agrobacterium*-infected plants (Singer et al., 2012). It is not known if T-circles represent a substrate or replication template for T-DNA integration, or whether they are a dead-end for T-DNA. Evidence from a small number of plant T-circles revealed that extra-chromosomal DNA end-joining occurs *via* a non-homologous pathway (Singer et al., 2012). This study suggested similarities in the mechanism involved in T-circle formation and T-DNA integration.

In this study, we sought additional evidence for similarities between these two processes. We investigated T-circle RB-LB junctions produced under different conditions, including in k*u80* plants, or generated using an *A. tumefaciensis virD2 ω* mutant. These T-circles were formed in *N. benthamiana* or in *Arabidopsis*. We determined the DNA sequence at T-circle RB-LB junction sites, the overall T-circle structure, and their rate of formation. Under all conditions tested, T-circles showed similar features to the structure of T-DNA molecules after integration into plant genomes, thus supporting the use of T-circles as a surrogate for T-DNA integration.

## Materials and Methods

### Bacterial Strains and Plasmids, and Plant Material

Plasmids and strains are described in Supplementary Table 7. We used *Escherichia coli* DH10B as the host for all cloning experiments. *Agrobacterium tumefaciens* EHA105 (Hood et al., 1993) was the host for most transformation experiments. To make a non-polar *virD2* mutant of EHA105, we first cloned a 7.2 kbp *Xho*I fragment containing the *virD* operon from pEHC13 into the *Xho*I site of pBluescript ks(+) to generate pE3332. We removed a 3.27 kbp blunted *Sph*I-*Xho*I fragment from pE3332 and cloned it into *Sma*I-*Xho*I digested pE3351 (pBluescript ks(+) lacking a *Kpn*I site) to make pE3353. We replaced the *Hin*dIII fragment of pE3353 with a 914 bp internal *Hin*dIII fragment of *vir*D2 from pE3052, generating pE3355. We removed an internal *Kpn*I fragment of pE3355 to make pE3356. We cloned an *Xho*I-*Not*I fragment containing the P*virD*-*virD1*-internal deletion *virD2*-*virD4* into the *Xho*I-*Not*I sites of pJQ200sk, generating pE3358. We electroporated pE3358 into *A. tumefaciens* EHA105, selecting for gentamicin resistance and sucrose sensitivity. We confirmed the resulting resolvent with a *virD2* deletion (At1697) by PCR. We linearized pUC18-P*virD*-*virD1*-ω substituted *virD2* (pE1500) with *Eco*RI, blunted it with Klenow fragment, and inserted it into the blunted *Pst*I site of pE1727, generating pE1745. We electroporated pE1745 into *A. tumefaciens* A136, generating At1132. We mated At1132 with E4 and screened for a strain (At1136) carrying pUC18-P*virD*-*virD1*-ω substituted *virD2* on the bacterial chromosome. We isolated pTiEHA105ΔvirD2 from At1697 and electroporated it into *A. tumefacjens* At1136, generating At1710. We removed pPH1JI from At1710, generating At1959.

The AMP-ORI and KAN-ORI T-DNA binary vectors were described previously (Singer et al., 2012). The TET-ORI T-DNA binary vector was constructed by PCR amplification of the *TetR* gene using the plasmid pSOUP as the template (Hellens et al., 2000) and primers TetR-EcoRI-F: 5′-atacgaattcctcatgtttgacagcttatcatcg-3′ and TetR-PstI-R: 5′-atacctgcagttcttggagtggtgaatccgttag-3′, and by PCR amplification of the *ColE1* ori region using the AMP-ORI plasmid as the template and primers ori322: PstI-F 5′-atacctgcagctcatgaccaaaatcccttaacgtgag-3′ and ori322-BamHI-EcoRI-R 5′-atacgaattcggatcccgtattgggcgctcttccgctt-3′. Restriction sites for *Pst*I and *Eco*RI at the 5′ ends of each primer pair were used to ligate the two fragments to generate a plasmid resistant to tetracycline. This plasmid was digested with *Bam*HI and *Eco*RI and ligated with the *Bam*HI-*Eco*RI backbone of the AMP-ORI binary vector pE4254 (pRCS11[10-amp]) to replace the ampicillin resistance gene and *ori* sequence to make pRCS11[TET-ORI (KS101, pE4252)]. This plasmid was further modified by deleting 65 bp between the *Bam*HI and *Pme*I sites at the RB side and replacing it with a 44 bp synthetic DNA sequence containing an I-*Sce*I site to make pRCS11[TET-ORI] (KS102, pE4253). The following antibiotics were used: For *E. coli*, ampicillin (100 μg/ml); kanamycin (50 μg/ml); and spectinomycin (50 μg/ml). For *A. tumefaciens*, spectinomycin (200 μg/ml); kanamycin (50 μg/ml); and rifampicin (10 μg/ml).

To make the T-DNA binary vector pE4636, we digested pUC19 with *Sac*I and *Sal*I and cloned it into the *Sac*I-*Sal*I site of pE4330, generating pE4579. We cloned a blunted *Sal*I fragment containing the *sacRB* gene from pE1961 into the BstZ171 site of pE4579, generating pE4636.

*Nicotiana benthamiana* plants were grown as previously described (Singer et al., 2012). *Arabidopsis thaliana* (ecotype Col-0) and the mutants *efr-1* (At5G20480; SALK_044334; Zipfel et al., 2006) and *ku80* (At1G48050; SAIL_714_A04) were used. The double mutant *ku80*/*efr-1* was generated by crossing these mutants and screening for homozygous double mutants.

### Transformation and T-Circle Isolation

T-circles were isolated from *N. benthamiana* leaves as previously described (Singer et al., 2012). T-circles from *Arabidopsis* were isolated by infecting seedlings using the AGROBEST method (Wu et al., 2014). Expression of β-glucuronidase (GUS) activity in *Arabidopsis* seedlings was measured to monitor transient transformation efficiency, using the T-DNA binary vector pBISN1 (Narasimhulu et al., 1996) and staining with 5-Bromo-4-chloro-3-indolyl-β-D-glucuronide (X-Gluc). *Nicotiana benthamiana* leaves were infiltrated using the *A. tumefaciens* wild-type or *virD2* mutant, discs were cut from infiltrated tissue, stained with X-gluc, and the intensity of staining quantified using ImageJ (US National Institutes of Health).

## Results

### Experimental Design

**Figure 1:**
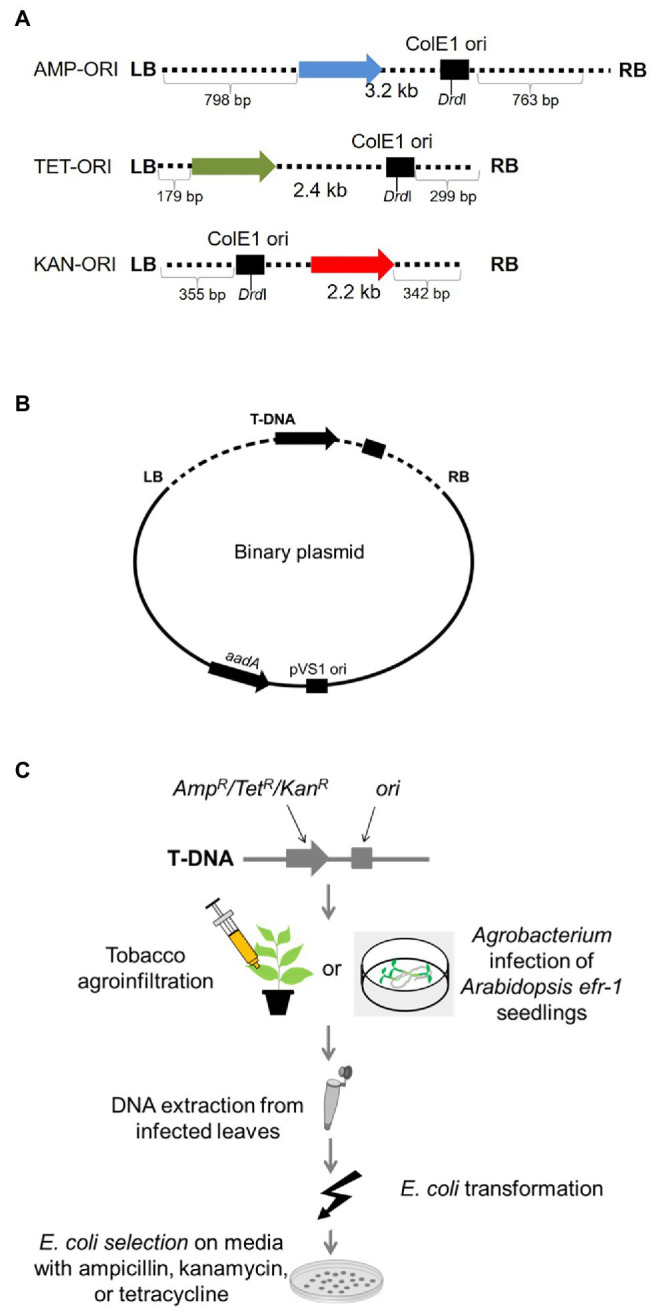
T-circle isolation using “simple” T-circle binary vectors. **(A)** Schematic diagram of the T-DNA regions of the various T-DNA binary vectors. AMP, TET, and KAN indicated genes encoding ampicillin, tetracycline, and kanamycin resistance, respectively. ORI indicates the ColE1 origin of replication. LB and RB indicate T-DNA left and right borders, respectively. **(B)** T-DNA binary vectors used in these experiments. aadA indicates a gene encoding spectinomycin resistance. pVS1 ori indicates the origin of replication of the plasmid in *Agrobacterium*. **(C)** Schematic diagram of the plant infection and T-circle isolation processes. *Nicotiana benthamiana* leaves are infiltrated or *Arabidopsis* seedlings are co-cultivated with *Agrobacterium tumefaciens* EHA105 harboring one of the T-circle binary vectors. After 3–6 days, total DNA is extracted from the plant tissue and used to transform *E. coli* cells. Transformants are selected on medium containing the antibiotic corresponding to the resistance gene in the T-DNA region, then later counter-screened on medium containing spectinomycin. Spectinomycin-sensitive colonies are progressed for T-circle characterization.

We first examined whether DNA patterns present at integrated T-DNA/plant DNA junctions can be found in T-DNA border junctions of T-circles. We recovered and sequenced numerous T-circles using different experimental conditions. For our initial experiments, we used T-DNA constructs containing the *ColE1* origin of replication and a bacterial ampicillin, tetracycline, or kanamycin resistance gene (Singer et al., 2012; this study), generating the T-DNA constructs AMP-ORI, TET-ORI, and KAN-ORI, respectively (Figure 1A). Binary vectors harboring these constructs were pRCS2 (for KAN-ORI) or pRCS11 (for AMP-ORI and TET-ORI), both of which contain in the plasmid backbone an *aadA* gene conferring bacterial spectinomycin resistance and the *pVS1* origin of replication for maintenance in *Agrobacterium* (Figure 1B; Singer et al., 2012; this study). *Agrobacterium tumefaciens* EHA105 containing these constructs were used to infect *N. benthamiana* and *Arabidopsis* (Figure 1C)*. Nicotiana benthamiana* was inoculated by leaf agroinfiltration (Singer et al., 2012). We could not obtain T-circles by *Arabidopsis* leaf agroinfiltration. Instead, we used the AGROBEST method and *Arabidopsis efr-1* mutant seedlings (Wu et al., 2014). Following infection, DNA was extracted and used for *E. coli* transformation. Colonies of transformed *E. coli* containing T-circles were identified based on the antibiotic resistance encoded by their T-DNA regions (ampicillin, tetracycline, or kanamycin) and their sensitivity to spectinomycin.

### RB-LB Junctions in Monomeric T-Circles

Previous studies of T-DNA/plant DNA junctions indicated that deletions occur more frequently and extensively at the T-DNA LB, and that the T-DNA RB is relatively more conserved (Tinland and Hohn, 1995; Tinland, 1996). In addition, microhomologies between T-DNA and plant DNA pre-integration sites often occur, especially near the LB (Windels et al., 2003; Tzfira et al., 2004; Muller et al., 2007; Kleinboelting et al., 2015; Gelvin, 2017, 2021). To examine if similar patterns exist in T-circles, we sequenced RB-LB junctions from T-circles involving a single T-DNA, referred to as monomeric T-circles. In monomeric T-circles the T-DNA RB side (the “head”) is ligated to the LB side (the “tail”). Complex T-circles may contain multiple T-DNA copies in various configurations, or large fragments of non-T-DNA regions (Singer et al., 2012).

**Figure 2:**
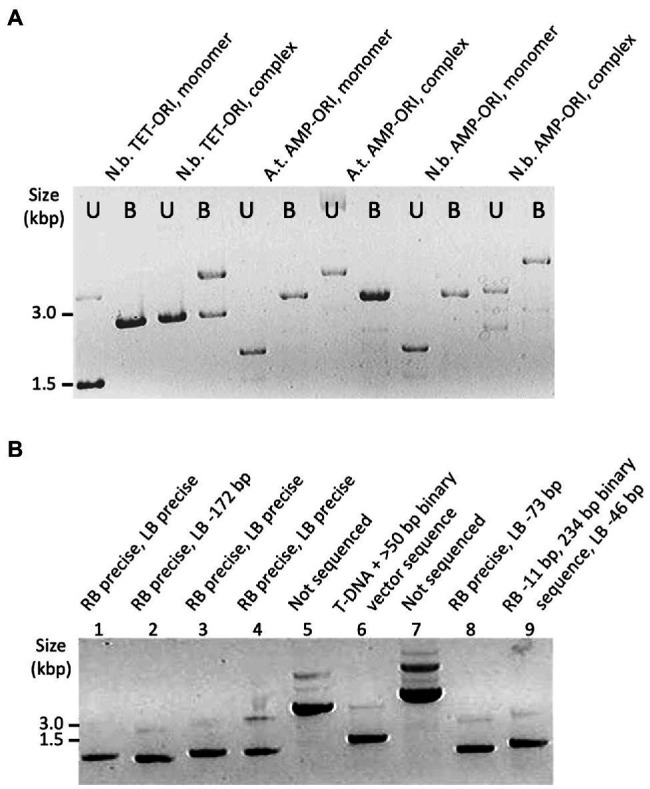
Examples of T-circle analyzed by agarose gel electrophoresis. **(A)** Samples of T-circles either undigested or *Bam*HI digested (U and B, respectively). T-circles were recovered from infected *Nicotiana benthamiana* or *Arabidopsis* plants (N.b. or A.t., respectively, above each lane) using the T-DNA binary vector TET-ORI or AMP-ORI. **(B)** Samples of T-circles (undigested) and the sequencing result for each junction above each lane. T-circles were isolated from *Nicotiana benthamiana* using the TET-ORI binary vector.

To identify monomeric T-circles, we screened non-digested plasmid DNA by electrophoresis through agarose gels (Figure 2). We also digested T-circle DNA with *Bam*HI, which cuts the T-DNA sequence once (Figure 2A). T-circles that were complex by this initial screening were excluded from DNA sequence analysis (Figure 2A: Lanes designated “complex,” and Figure 2B: Lanes 5 and 7). The remainder of the T-circles were sequenced at the junction between the T-DNA RB and LB regions. In several cases DNA sequencing revealed junctions that contained fragments of DNA which are not part of T-DNA. If such fragments were larger than 50 bp, these T-circles were re-classified as complex (Figure 2B; Lanes 6 and 9; Supplementary Figure 1).

Monomeric T-circles represented 65% (N = 211) of those recovered from *N. benthamiana* (61% of TET-ORI and 68% of AMP-ORI T-circles) and 98% (*N* = 130) of those isolated from *Arabidopsis efr-1* (Supplementary Table 1). Thus, T-circles isolated from *N. benthamiana* were more frequently complex than were those formed in *Arabidopsis*.

Sequence analysis of T-DNA junctions from monomeric T-circles showed a prevalence of conserved VirD2 cleavage positions between nucleotides three and four of the borders (Wang et al., 1987). Such “precise” ends generally occurred more often at the RB side of T-DNA. Using *N. benthamiana* as a host, precise ends occurred in 81% of the RBs and in 48% of the LBs (Table 1, Supplementary Table 2). Using *Arabidopsis efr-1*, precise ends occurred in 95% of the RBs and in 92% of the LBs (Table 1). Thus, precise ends were more prevalent in T-circles isolated from *Arabidopsis* than from *N. benthamiana*. Whereas precise RBs were frequently ligated to deleted LBs, among 139 precise LBs 138 were ligated to precise RBs (38 precise LBs from *N. benthamiana* and 101 precise LBs from *Arabidopsis efr-1*).

**Table 1:** Summary of extent of deletions at the RB and LB ends of RB-LB junctions of monomeric T-circles from *Nicotiana benthamiana* and *Arabidopsis thaliana* infected with EHA105.

Deletion at T-DNA end (bp)							
	Precise	1	2	3–10	11–100	>100	Number of junctions sequenced
*Nicotiana benthamiana*							
RB ends	65	3	4	2	4	2	80
LB ends	38	1	0	1	22	18	80
*Arabidopsis thaliana*							
Col-0							
RB ends	10	0	0	0	0	0	10
LB ends	10	0	0	0	0	0	10
*efr-1*							
RB ends	106	2	0	1	1	1	111
LB ends	102	0	0	3	5	1	111

Deletions at RB ends were mostly fewer than 10 nucleotides, and often only 1 or 2 nucleotides. In contrast, most deletions at LBs involved more than 10 nucleotides (Table 1, Supplementary Tables 2 and 3). The maximum number of nucleotides deleted from T-DNA ends in T-circles derived from these binary vectors is restricted by the positions of the *ori* and antibiotic resistance genes as these elements are required to recover T-circles (Figure 1). Below, we describe a different T-circle binary vector that can support recovery of T-circles with larger deletions.

### RB-RB and LB-LB Junctions in Heterodimer T-Circles

Multiple T-DNA molecules often integrate adjacent to each other. We therefore examined RB and LB junctions from T-circles that are made from two different T-DNAs (T-DNA heterodimers).

**Figure 3:**
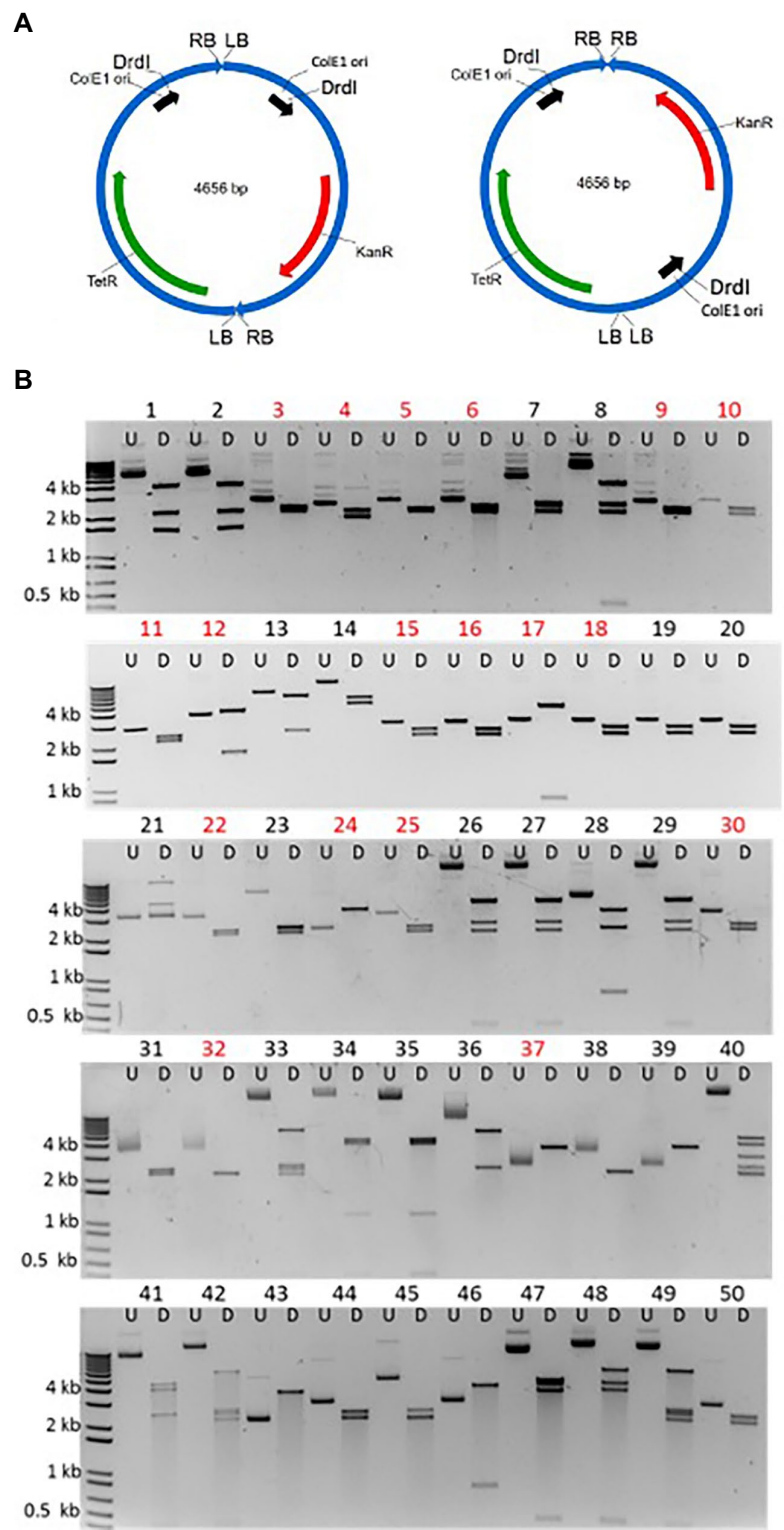
Characterization of T-circles composed of two different T-DNAs. **(A)** Two T-DNAs can end-join to each other in either of two configurations: “Head-to-tail” that will produce 3.9 and 0.7 kbp *Drd*I fragments (left) or “head-to-head” and “tail-to-tail” that will produce 2.2 and 2.4 kbp *Drd*I fragments (right). **(B)** 50 T-circles conferring resistance to both kanamycin and tetracycline following co-agroinfiltration with *Agrobacterium tumefaciens* EHA105 containing the KAN-ORI and TET-ORI T-DNA binary vectors. Each plasmid is shown undigested (U) and *Drd*I digested (D). Eighteen unique sequenced T-circles are marked in red (T-circles #19, #20, #32, #38, #39, #43, #44, and #50 are duplicates).

Figure 3A schematically shows the two possible arrangements of T-circle heterodimers, which can be arranged “head-to-tail” (RB-to-LB) or “head-to-head/tail-to-tail” (RB-to-RB/LB-to-LB). To isolate these heterodimeric T-circles, we co-infiltrated *N. benthamiana* leaves with two *Agrobacterium* strains containing T-circle TET-ORI or KAN-ORI binary vectors, followed by selection of *E. coli* colonies on medium containing both tetracycline and kanamycin. We isolated 50 such T-circle heterodimers. To reveal the size and configuration of T-DNAs constituting these T-circles, we determined the sizes of their *Drd*I restriction endonuclease fragments. Because of potential difficulties in sequencing T-circles made up of more than two T-DNAs, we excluded from our analyses T-circles whose size is greater than that expected from TET-ORI and KAN-ORI heterodimers (4.6 kbp; Figure 3B). DNA sequence analysis of the junctions from these remaining 26 T-circles revealed that 18 were unique, whereas eight were experimental duplicates. From these 18, 16 were arranged “head-to-head”/“tail-to-tail.” Only one T-circle was arranged “head-to-tail” (Figure 3B T-circle #17). The remaining sequenced T-circle (#12) contained a third short T-DNA fragment (Supplementary Figure 2). Supplementary Figure 3 schematically shows the sequencing results of the various heterodimeric T-circles.

DNA sequence analysis of 32 RBs (from 16 RB-RB junctions) and 28 LBs (from 14 LB-LB junctions) revealed patterns similar to those found at RB-LB junctions of monomeric T-circles (Table 2 and Supplementary Table 4). Among RB-RB junctions 75% of the RBs were precise, similar to 81% of the RBs that were precise in RB-LB junctions. However, none of the LB-LB junctions were precise, in contrast to 48% of the LBs that were precise in RB-LB junctions. In three RB-LB junctions between KAN-ORI and TET-ORI T-circle heterodimers (two in #17 and one in #12), both the LB and RB ends were precise (Supplementary Table 4).

**Table 2:** Summary of extent of deletions in T-circle heterodimers at the RB and LB ends of RB-RB and LB-LB junctions isolated from *Nicotiana benthamiana*.

Deletion at T-DNA end (bp)							
	Precise	1	2	3–10	10–100	>100	Number of junctions sequenced
RB ends	24	4	2	0	1	1	32
LB ends	0	0	0	0	22	6	28

### Microhomology and Filler DNA in T-Circles

Microhomologies between integrated T-DNA sequences and plant pre-integration site sequences at DNA junctions have frequently been observed and associated with repair of DNA double strand breaks (Gorbunova and Levy, 1997; Windels et al., 2003; Tzfira et al., 2004; Muller et al., 2007; Kleinboelting et al., 2015; Gelvin, 2017, 2021). Therefore, we examined patterns of microhomologies at DNA junctions in T-circles.

It should be noted that 12 bp of microhomology can be alleged in all RB-LB junctions with precise ends at both sides. However, the DNA region involved in such potential microhomology resides outside the boundaries of T-DNA from the RB side. Therefore, this microhomology can be involved only if a read-though of the RB had occurred during T-DNA processing in *Agrobacterium*. However, in one T-circle (#022-7) a precise RB and LB were separated by five nucleotides of filler DNA (Supplementary Table 3), suggesting that a RB read-through microhomology region is not required for precise RB and LB ends.

Microhomologies were frequent if T-DNA ends were deleted. In RB-LB junctions with deletions at both ends, microhomologies of 1 to 4 nucleotides were present in 6 of 19 junctions (Supplementary Tables 2 and 3). In LB-LB junctions with deletions at both ends, microhomologies of 1 to 6 nucleotides were present in 13 of 14 junctions. In RB-RB junctions all 16 junctions had at least one precise end, with deletions of mostly 1 or 2 nucleotides. Seven of 16 RB-RB junctions contained microhomologies of 1 or 2 bp, but at least three of these are likely not true microhomologies as they are possible only if a read-thorough of a precise RB occurred during T-DNA processing in *Agrobacterium* (Supplementary Table 4). Overall, LBs show a higher degree of use of microhomologies, similar to what has been reported in T-DNA/plant DNA junctions of integrated T-DNAs (Windels et al., 2003; Muller et al., 2007; Kleinboelting et al., 2015).

Filler DNA, defined as short DNA sequences from sources other than sequences directly at the T-DNA or plant DNA junction site, may occur at T-DNA/plant genome junctions. T-circles may contain filler DNA, mostly 1 to 5 nucleotides in 24 cases (Table 3). Among 216 junctions where both the RB and LB were precise, only one included filler DNA. On the other hand, filler DNA was more frequent at junctions involving deleted T-DNA ends. Filler DNA was present in 13 of 36 junctions involving a precise RB with a deleted LB and in 6 of 20 junctions involving deleted RB and LB ends. Filler DNA was also found in LB-LB junctions and RB-RB junctions. Notably, all 16 filler DNAs next to precise RBs were either A or T nucleotides (Table 3).

**Table 3:** Filler DNA from unknown source at T-DNA junctions.

T-DNA junction	Number of Junctions	Junctions with filler DNA	Filler DNA
Precise RB-Precise LB	216	1	TAATA
Precise RB-Deleted LB	36	13	T, T, A, A, AAAA, T, A, T, A, A, A, T, A
Deleted RB-Deleted LB	20	6	A, GT, AGCT, G, A, GTC
Deleted RB-Precise LB	1	1	TTAATAGTTTAAACTGAAGCGCAGAT
Precise RB- Precise RB	9	2	ATA, A
Precise RB- deleted RB	8	0	
Deleted LB-Deleted LB	14	1	A

### Plant DNA, *Agrobacterium* Chromosomal DNA, and *Agrobacterium* Plasmid DNA in T-Circles

Most filler DNAs at T-DNA/plant genome junctions comprise short sequences of T-DNA or sequences from the binary vector backbone (Simpson et al., 1982; Kononov et al., 1997; Wenck et al., 1997). Some T-DNA insertions may include plant DNA sequences from sites different from the site of insertion (Kleinboelting et al., 2015). DNA from both the *Agrobacterium* chromosomes and from other *Agrobacterium* replicons (Vir helper plasmids, the “cryptic” plasmid pAtC58, non-T-DNA sequences of the Ti-plasmid, etc.) have been reported at T-DNA insertion sites (Ulker et al., 2008; Nishizawa-Yokoi et al., 2021).

Some of the sequenced T-circles contained additional DNA between the sequenced borders (Table 4; Supplementary Figure 3). The DNA sequences in these T-circles was in many cases homologous to internal T-DNA regions or to the binary vector backbone, as previously reported (Singer et al., 2012). In addition, a 375 bp fragment of *Agrobacterium* chromosomal DNA was found in T-circle #052-44, whereas larger fragments of *Agrobacterium* Ti-plasmid DNA were found in T-circles #052-22 and #052-66. *Nicotiana benthamiana* DNA was found in T-circles #008-73 and #052-18 (290 bp and 188 bp, respectively). Thus, different types of DNA, that have previously been identified at T-DNA/plant DNA junctions in transgenic plants, can also be found between T-circle borders.

**Table 4:** Sequenced T-DNA junctions of complex T-circles.

Sample no.	Strain	Construct	RB*	Microhomology^a^	Filler DNA^b^	Microhomology^c^	LB^*^
*Nicotiana benthamiana*							
#002–23	EHA105	TET-ORI	Readthrough >380 bp	NA	NA	NA	NA
#003–50	EHA105	TET-ORI	Precise	2 (GA)	236 bp binary +9 bp internal T-DNA sequence	0	−72
#003–61	EHA105	TET-ORI	−1	2 (TG)	>486 bp binary sequence	NA	NA
#003–62	EHA105	TET-ORI	Precise	4 (TTGA)	>489 bp binary sequence	NA	NA
#003–64	EHA105	TET-ORI	Precise +1	0	>202 bp internal T-DNA sequence	NA	NA
#008–73	EHA105	TET-ORI	Precise	1 (A)	290 bp plant DNA	6 (TCAGGC)	−11
#009–12	EHA105	TET-ORI	Precise +4	5 (CAGGC)	>585 bp binary sequence	NA	NA
#050–14	EHA105	AMP-ORI	Readthrough 151 bp	NA	0	0	−215
#050–23	EHA105	AMP-ORI	Precise	0	>431 bp binary sequence	NA	NA
#052–9	EHA105	AMP-ORI	Precise+1	1 (C)	284 bp binary sequence	3 (AGC)	−456
#052–17	EHA105	AMP-ORI	Readthrough 224 bp	NA	0	0	−237
#052–18	EHA105	AMP-ORI	−21	4 (TTCA)	188 bp plant DNA	0	−239
#052–22	EHA105	AMP-ORI	Precise +1	0	>406 bp Ti plasmid sequence	NA	NA
#052–28	EHA105	AMP-ORI	Precise	0	84 bp T-DNA sequence	3 (GCT)	−436
#052–35	EHA105	AMP-ORI	Precise	0	>484 bp binary sequence	NA	NA
#052–44	EHA105	AMP-ORI	Precise+1	1 (C)	375 bp *Agrobacterium* chromosomal DNA	3(CGA)	−317
#052–45	EHA105	AMP-ORI	Readthrough 129 bp	NA	0	3 (TTT)	−714
#052–55	EHA105	AMP-ORI	Precise	2 (GA)	74 bp internal T-DNA sequence	2(GG)	−683
#052–60	EHA105	AMP-ORI	Precise +2	2 (CA)	>560 bp binary sequence	NA	NA
#052–63	EHA105	AMP-ORI	Readthrough 192 bp	NA	0	0	−265
#052–66	EHA105	AMP-ORI	Precise	0	>799 bp Ti plasmid sequence	NA	NA
*VirD2ω*							
#005–8	At1959	TET-ORI	−11	0	234 bp binary sequence	4 (AACA)	−46
*Arabidopsis Col-0*							
#021–4	EHA105	AMP-ORI	Readthrough >461 bp	NA	NA	NA	NA

NA, not available.

* Right border (RB) and left border (LB) numerical values represent the position in the DNA relative to the precise end.

a Microhomology between RB and filler DNA.

b Filler DNA is defined here as any DNA sequence not part of the contiguous T-DNA.

c Microhomology between filler and LB.

### Generation of T-Circles Using an Improved T-circle Binary Vector

The T-circle binary vectors used for our initial experiments all contain a small T-DNA region with only sequences important for replication and antibiotic selection in *E. coli*. Because both of these elements are essential for T-circle rescue, large T-DNA border deletions could not be tolerated. Extensive time-consuming counter-screening was required to differentiate between true T-circles and contaminating binary vector sequences, which represent the large majority of antibiotic-resistant *E. coli* transformants.

**Figure 4:**
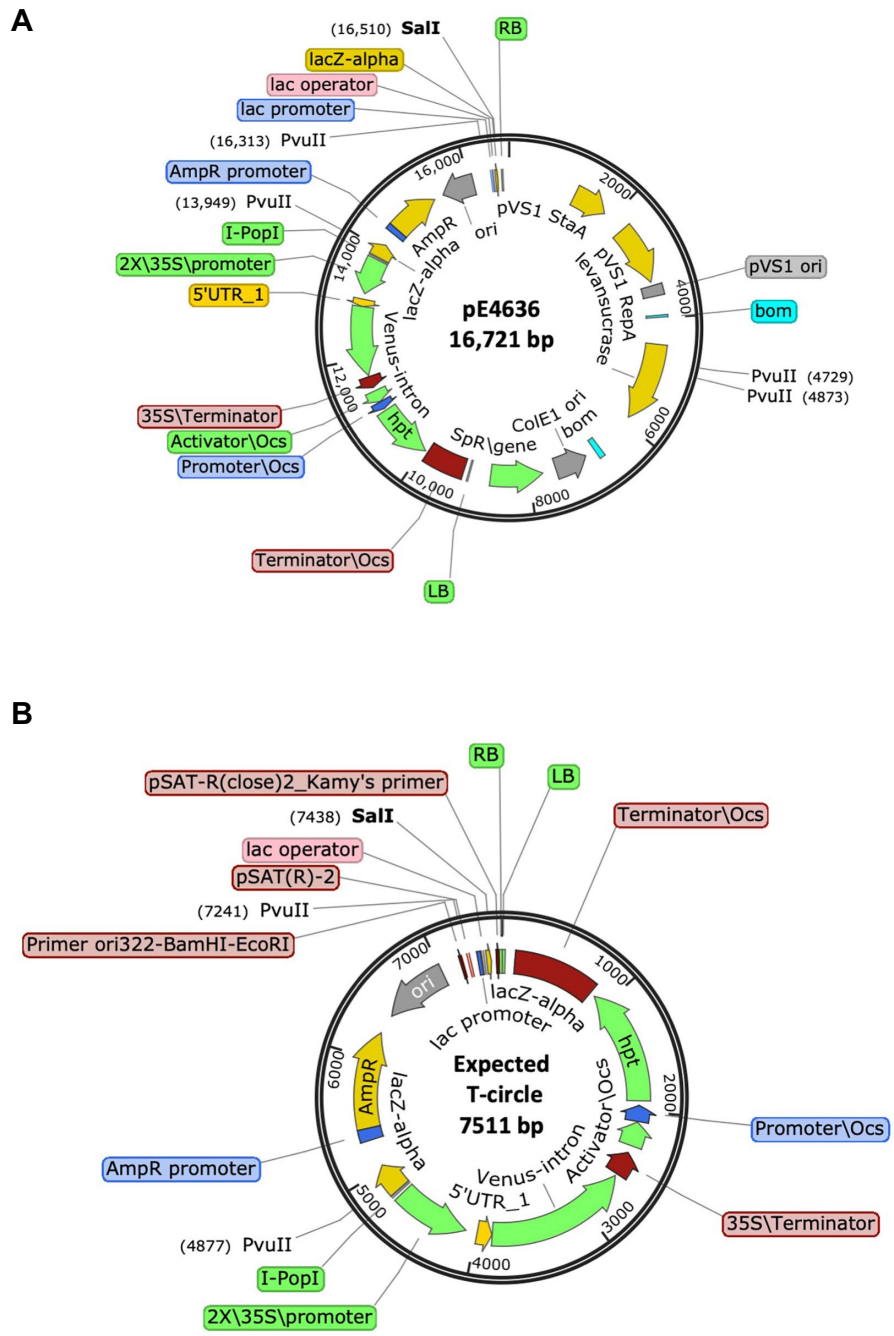
Schematic representation of **(A)** The T-DNA binary vector pE4636, and **(B)** The expected T-circle formed from this plasmid by joining of the T-DNA right and left borders (RB and LB, respectively).

We therefore constructed a new T-circle binary vector (pE4636) to ameliorate these problems. The new plasmid contains within the T-DNA region a *ColE1 ori* sequence and a *β-lactamase* (ampicillin-resistance) gene. We positioned a plant-active *Venus-intron* gene next to the *β-lactamase* gene, and a plant-active *hptII* gene next to the LB. We also placed within the vector backbone sequence a *sacB* gene. This new T-circle binary vector allowed us to monitor plant transient transformation (Venus fluorescence) and stable integration of T-DNA into the plant genome (hygromycin resistance). The *sacB* gene allowed for negative selection of transformed *E. coli* cells containing the entire binary vector rather than T-circles (sucrose sensitivity). Importantly, the *hptII* and *Venus-intron* genes near the LB allowed for detection of large LB deletions without disrupting sequences essential for T-circle recovery in *E. coli*. Figure 4 shows maps of this new binary vector and the full-size T-circle that it could generate.

We used this new T-circle binary vector to obtain T-circles from infiltrated *N. benthamiana* leaves. Isolated T-circles were first digested with *Sal*I to determine their size and whether sequences more than 76 bp from the RB remained intact. We used *Pvu*II digestion to determine if DNA sequences more than 273 bp from the RB remained intact. If either of these sites remained intact, we sequenced across the RB-LB interface using primers set back from the RB. If neither of these two sites existed, we subjected the T-circle plasmids to WideSeq analysis to determine their entire sequence.

Of the 42 T-circles characterized, sequences extending to and through the RB region were generated by Sanger sequencing for 30 T-circles. An additional 12 T-circles were completely sequenced using WideSeq. The data are summarized in Table 5 and in Supplementary Table 5. In total, 23 T-circles (55%) contained a precise RB, whereas none contained a precise LB. Microhomology was found in 13 T-circles (31%) at the RB region and seven T-circles (17%) at the LB region. The LB region frequently showed long deletions, extending from a few hundred bases to >5.9 kbp. Long regions of filler DNA were found in nine T-circles (21%), and an additional four T-circles contained a few bp of filler DNA between the borders. These filler DNAs derived from *N. benthamiana*, the “cryptic” plasmid pAtC58, or from the binary vector (Supplementary Table 5). More than one third of the T-circles (15; 36%) contained major rearrangements, including binary vector or T-DNA sequences in an inverted orientation, or other major sequence rearrangements of unknown origin.

**Table 5:** Summary of properties of T-circles from *Nicotiana benthamiana* generated by pE4636.

Number	Precise RB	Precise LB^a^	Microhomology at RB region	Microhomology at LB region	Filler DNA	Major rearrangements
42	23 (55%)	0 (out of 39; 0%)	13 (31%)	7 (17%)	2 (plant; 5%) 3 (pAtC58; 7%) 4 (binary; 10%) 4 (few bp; 10%)	Binary vector or T-DNA reverse complement or other unknown rearrangement (15; 36%)

a In some instances, Sanger sequencing did not extend far enough to obtain a LB region sequence if there were a large filler. For some plasmids, Wide-seq analysis revealed the entire T-circle sequence.

T-circles derived from the new T-DNA binary vector resembled those derived from the simpler binary vector. However, the long “buffer” of T-DNA sequences adjacent to the LB allowed us to recover large LB deletions and sequence rearrangements.

### Ku80 Is Not Required for T-Circle Formation in Plants

We tested if the classical NHEJ pathway were involved in T-circle formation by examining the rate of T-circle formation and patterns of DNA end-joining at junctions of T-circles in an *Arabidopsis ku80/efr-1* double mutant. The *efr-1* mutant allows higher transient transformation of *Arabidopsis* (Zipfel et al., 2006).

*Arabidopsis ku80/efr-1* and control *efr-1* seedlings were infected with *Agrobacterium* containing the AMP-ORI construct. DNA was extracted and used for *E. coli* transformation. Under our conditions, 82 of 516 (15.9%) and 75 of 475 (15.8%) of the amp-resistant *E. coli* colonies isolated from *efr-1* and *ku80*/*efr-1* plants, respectively, were sensitive to spectinomycin, as expected from colonies that contain T-circle, rather than binary vector, DNA. Therefore, Ku80 deficiency did not affect the rate of T-circle formation. We sequenced 63 RB-LB junctions from T-circles recovered from *ku80/efr-1* plants (Supplementary Table 6). Most junctions at both the LB and RB were precise, as was found in T-circles recovered from control *efr-1* plants. Therefore, Ku80 deficiency did not result in differences in either the rate of formation or T-DNA border patterns of T-circles.

### T-Circles Generated Using an *Agrobacterium* VirD2 *ω* Mutant Form Less Frequently Than Do T-circles Generated Using a Wild-Type *Agrobacterium* Strain

**Figure 5:**
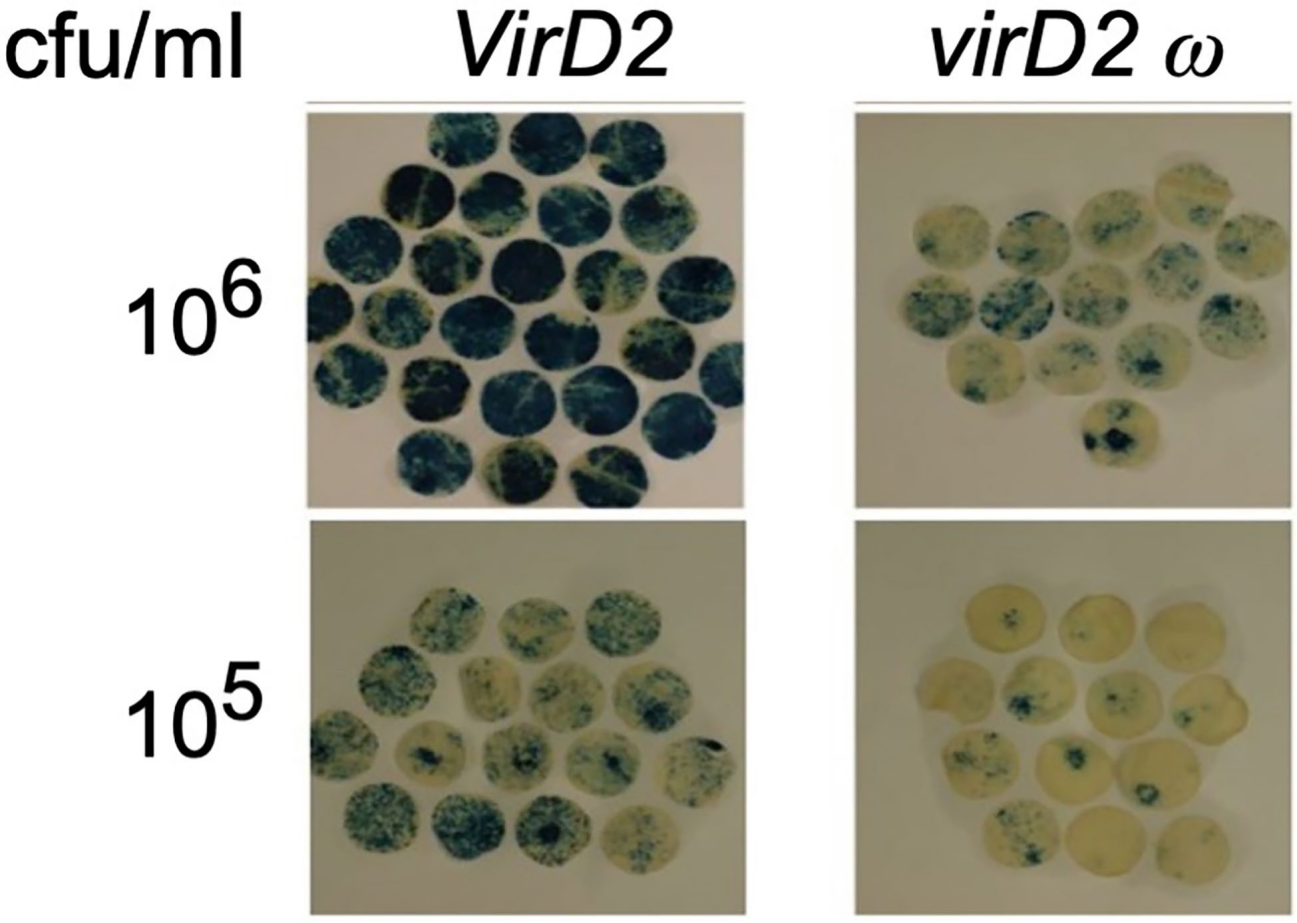
Transient expression of GUS activity in *N. benthamiana* leaves agroinfiltrated with *Agrobacterium* containing a wild-type *VirD2* gene (left panels) or the *virD2 ω* mutant (right panel). *Agrobacterium tumefaciens* containing a wild-type *virD2* gene, the AMP-ORI T-circle binary vector, and the T-DNA binary vector pBISN1 or a similar strain with the *ω* mutant *virD2* gene were infiltrated at the indicated concentration. Discs cut from leaves after 4 days were stained with X-gluc, cleared with 70% ethanol, and the relative staining intensity calculated using ImageJ software. cfu, colony forming units.

A substitution mutation in the omega (ω) domain of VirD2 (Shurvinton et al., 1992) severely reduces (>95%) T-DNA integration while only reducing T-DNA transfer into plant cells by ~75–80% (Mysore et al., 1998). We generated an *A. tumefaciens* EHA105 derivative (At1959) that contains this *virD2* mutation. We first indirectly examined the rate of T-DNA transfer by this mutant strain by measuring transient β-glucuronidase (GUS) expression, conferred by a *gusA-*intron gene within T-DNA (pBISN1). We agroinfiltrated *N. benthamiana* leaves with wild-type (At2120) and mutant *Agrobacterium* (At2121 or At2162) at low cell density (~10^6^ and ~ 10^5^ cfu/ml) to avoid a saturation response (Figure 5). T-DNA transfer from the *virD2 ω* mutant was 7.5–9.1% that of T-DNA transfer from the wild-type *VirD2* strain.

We next infiltrated *N. benthamiana* leaves with *Agrobacterium* strains harboring the AMP-ORI or TET-ORI T-DNA binary vectors. Infection using the wild-type strain resulted in 144 of 1,118 (12.9%) colonies that were resistant to ampicillin or tetracycline but sensitive to spectinomycin. However, using the *virD2 ω* mutant only 12 of 3,450 (0.35%) of the transformed *E. coli* colonies were resistant to ampicillin or tetracycline but sensitive to spectinomycin. Therefore, the rate of T-circle formation obtained using the *virD2 ω* mutant *Agrobacterium* strain was 2.7% (0.35/12.9) of that obtained using *Agrobacterium* strains with a wild-type *VirD2* gene.

DNA sequence analysis of RB-LB junctions showed that LB T-circle sequences derived from inoculation with the *virD2 ω* mutant were similar to those obtained using a wild-type *VirD2 Agrobacterium* strain (Table 6). However, RB sequences from six T-circles derived from use of the *virD2 ω* mutant strain were all precise, as opposed to our previous finding of only 81% precise RBs using a wild-type *Agrobacterium* strain. To obtain more examples of T-circles generated using the *virD2 ω* mutant *Agrobacterium* strain, we infiltrated *N. benthamiana* leaves with *A. tumefaciens* At2332, a *virD2 ω* mutant (At1959) containing the new T-circle binary vector pE4636. DNA sequence analysis of 17 T-circle RB-LB junctions again indicated only precise RBs. In addition, LBs in T-circles generated by the *virD2 ω* mutant *Agrobacterium* strain were more frequently precise than those obtained using *Agrobacterium* strains containing a wild-type *VirD2* gene (42% vs. 0%; Tables 5 and 6). Thus, the VirD2 *ω* mutation altered the use of both RBs and LBs in T-circles, indicating that VirD2 is involved in T-circle formation.

**Table 6:** T-DNA junctions of T-circles from *Agrobacterium benthamiana* using *virD2 ω* mutant *Agrobacterium* strains.

Number characterized	Strain	Construct	Precise RB	Precise LB^a^	RB Microhomology	LB Microhomology^a^	Filler DNA
6	At1959	TET-ORI or AMP-ORI	6 (100%)	3 (50%)	1 (17%)	0	1 (1 bp, A)
18	At2332	pE4636	18 (100%)	5 (of 12; 42%)	5 (28%)	2 (of 13; 15%)	6 (pAtC58) 3 (linear chromosome)

a In some instances, Sanger sequencing did not extend far enough to obtain a LB region sequence if there were a large filler. For some plasmids, Wide-seq analysis revealed the entire T-circle sequence.

### T-Circles Form *in planta* but Not in *Agrobacterium*

We previously showed that T-circles cannot be recovered when plants are infiltrated with a *virB* mutant *Agrobacterium* strain, indicating that T-circles are formed *in planta*. We also showed that T-circles do not result from ligation of T-DNA molecules after transformation into *E. coli* (Singer et al., 2012). Two studies had previously indicated that circular T-DNA molecules could form in *Agrobacterium* as a result of recombination between similar sequences in the T-DNA LB and RB regions (Koukolikova-Nicola et al., 1985; Machida et al., 1986). To test whether the T-circles we observed had formed by recombination in *Agrobacterium*, we investigated T-circle heterodimer formation in *Agrobacterium*. *Agrobacterium* strains individually containing the KAN-ORI or TET-ORI binary vectors were co-infiltrated into *N. benthamiana* leaves. After 6 days, 50 mg of plant tissue was crushed in sterile LB medium and plated onto solidified YEP medium containing either spectinomycin, kanamycin, tetracycline, or kanamycin plus tetracycline. *Agrobacterium* colonies appeared on YEP medium containing kanamycin or tetracycline, but not on medium containing both kanamycin and tetracycline. Furthermore, we tested colonies that grew on tetracycline (*n* = 158), kanamycin (*n* = 102), or spectinomycin (*n* = 275). None of these colonies grew on medium containing both kanamycin and tetracycline. These results indicate that recombination between the KAN-ORI and TET-ORI T-DNA regions did not occur in *Agrobacterium*.

To show that T-circle heterodimers had formed *in planta* during these infiltrations, total DNA was extracted from co-Agroinfiltrated leaves and used to transform *E. coli*. From 192 colonies selected on kanamycin, 64 were spectinomycin-sensitive, indicating T-circle formation. Of these, seven were also tetracycline-resistant, indicating T-circle heterodimer formation. Similarly, from 190 colonies selected on tetracycline, 42 were spectinomycin-sensitive and seven of these were also kanamycin-resistant, again indicating T-circle heterodimer formation. Thus, in total 4.7% of the *E. coli* colonies selected on either kanamycin or tetracycline were resistant to both antibiotics.

The results of these experiments indicate that we could recover T-circle heterodimers from Agro-infiltrated *N. benthamiana* leaves but could not detect recombination between these same T-DNA regions in *Agrobacterium* cells.

## Discussion

Nicking of the T-DNA border sequences by VirD2 releases a T-strand containing border nucleotides 1–3 at the RB and nucleotides 4–25 at the LB. Before or during the process of T-DNA integration into the plant genome, alterations of T-DNA commonly occur. These include resection of T-strands at the LB and/or RB ends. In addition, filler DNA may occur at T-DNA/plant DNA junctions; this filler may come from within T-DNA or from the plant genome (Kleinboelting et al., 2015). Filler DNA may also originate from other *Agrobacterium* replicons, including bacterial chromosomal DNA or other bacterial plasmids (Ulker et al., 2008; Nishizawa-Yokoi et al., 2021). Microhomologies between T-DNA border region sequences and plant pre-integration sequences may occur; these are generally more prevalent near the LB (Windels et al., 2003; Tzfira et al., 2004; Muller et al., 2007; Kleinboelting et al., 2015; Gelvin, 2017, 2021). T-DNA may integrate into plant DNA as a monomer, as dimers (RB-to-LB [head-to-tail], RB-RB [head-to-head], or LB-LB [tail-to-tail]), or as multimers. Finally, integration of T-DNA frequently causes major plant chromosome rearrangements (Castle et al., 1993; Nacry et al., 1998; Tax and Vernon, 2001; Lafleuriel et al., 2004; Curtis et al., 2009; Clark and Krysan, 2010; Majhi et al., 2014; Ruprecht et al., 2014; Hu et al., 2017; Jupe et al., 2019). These different DNA patterns at T-DNA/plant DNA junctions comprise the foundations for models explaining possible mechanisms of T-DNA integration (reviewed in Tzfira et al., 2004; Gelvin, 2017, 2021; Singer, 2018).

The mechanism of T-DNA integration into the plant genome, and whether various plant DNA repair pathways and specific proteins play roles in it, remains highly controversial, with many different theories and conflicting results. Homologous recombination is not used for T-DNA integration; despite large regions of homology between sequences within T-DNA and plant DNA, targeting using homology is extremely rare in plants, although it is common in yeast (Rolloos et al., 2014, 2015). Among the non-homologous end-joining (NHEJ) pathways, both the “classical” (Ku-dependent; cNHEJ) and the microhomology-mediated (MMEJ) end-joining pathways have been suggested to promote T-DNA integration (Mayerhofer et al., 1991; Tinland and Hohn, 1995; Tinland, 1996; Tzfira et al., 2004; van Kregten et al., 2016; Gelvin, 2017, 2021). Genetic experiments to determine T-DNA integration pathway components have involved ablation of specific cNHEJ and/or MMEJ components, followed by quantitation of stable transformation frequencies, usually by antibiotic/herbicide selection of transgenic events. Some of these studies reported a decrease in stable transformation using *ku70/80* or DNA ligase IV (*lig4*) mutants, suggesting involvement of the cNHEJ pathway in T-DNA integration (Friesner and Britt, 2003; Li et al., 2005; Jia et al., 2012; Mestiri et al., 2014; Saika et al., 2014). Other studies showed little or no difference among stable transformation frequencies using cNHEJ or MMEJ mutants (Gallego et al., 2003; van Attikum et al., 2003). Still other studies showed that mutation or down-regulation of *ku70/80*, *xrcc4*, or DNA ligase VI (*lig6*) increased the frequency of stable transformation (Vaghchhipawala et al., 2012; Park et al., 2015). Most of these studies suffered from the limitations of using stable transformation (with selection) as a proxy for T-DNA integration (see discussion in Gelvin, 2021). However, some studies examined T-DNA integration biochemically in the absence of selection (Vaghchhipawala et al., 2012; Park et al., 2015; Nishizawa-Yokoi et al., 2021). These latter studies indicated that mutation of cNHEJ and MMEJ genes did not substantially decrease the amount of T-DNA integrated into the plant genome and may, in some instances, increase it. The initially reported requirement for DNA polymerase θ, an essential component of MMEJ, to obtain stable transformants (van Kregten et al., 2016) has been disputed (Nishizawa-Yokoi et al., 2021). Thus, the participation of various NHEJ pathways and individual components of these pathways remains highly controversial, and genetic approaches to solving this conundrum may be limited if proteins required for T-DNA integration are essential for cellular viability (Gelvin, 2021).

We propose that studying the formation of T-circles will inform us about T-DNA integration. However, we first needed to show that T-circle border junctions resembled T-DNA/plant DNA junctions, and that factors which influence T-DNA/plant DNA junctions similarly influence T-DNA border junctions in T-circles. We therefore sequenced hundreds of T-circle border junctions, generated in both wild-type *N. benthamiana* and in *Arabidopsis*, and generated in an *Arabidopsis ku80* cNHEJ mutant. We also examined the amount of T-circles formed and the precision of LBs and RBs following infection by an *Agrobacterium* VirD2 *ω* mutant. The results of these studies indicated that in all aspects examined, T-circle border junctions resembled what has been well documented with T-DNA/plant DNA junctions.

### The Structure of T-DNA Border Junctions in T-Circles Resembles T-DNA/Plant DNA Junctions

Within *Agrobacterium*, T-strands retain, at the LB, nucleotides 4–25 of the 25 bp border repeat, whereas the RB contains nucleotides 1–3 covalently linked to VirD2 (Wang et al., 1987; Ward and Barnes, 1988; Young and Nester, 1988; Durrenberger et al., 1989; Howard et al., 1989). A “simple” and “precise” integration of T-DNA into plant DNA would result in plant DNA joined to one T-DNA molecule, using nucleotides 4–25 at the LB and nucleotides 1–3 at the RB. Such ideal T-DNA insertions are rarely observed. Rather, multiple copies of T-DNA often integrate next to each other in RB-LB, RB-RB, or LB-LB orientation (Windels et al., 2003; Muller et al., 2007). Deletions of plant DNA at the integration site frequently exist, and T-DNA deletions at the borders are common. T-DNA border deletions are especially prevalent and may be large at the LB, which is not protected by VirD2, but may also occur at the RB (Durrenberger et al., 1989; Windels et al., 2003; Kleinboelting et al., 2015). “Filler” DNA may appear at the borders. This filler DNA may be from within T-DNA, from other regions of the Ti-plasmid (or the binary vector backbone), *Agrobacterium* chromosomal DNA, or DNA from other *Agrobacterium* replicons (Muller et al., 2007; Ulker et al., 2008; Nishizawa-Yokoi et al., 2021). Filler DNA from the plant genome usually derives from sequences nearby, or on the same chromosome, as the T-DNA integration site, although sequences from other chromosomes may be used (Kleinboelting et al., 2015). Finally, microhomology between T-DNA borders (or deleted borders) and sequences immediately upstream of the integration site is frequent, especially at the LB (Windels et al., 2003; Muller et al., 2007; Kleinboelting et al., 2015). This resulting picture of junctions at T-DNA integration sites suggests the use of microhomology to “copy in” other DNA sequences by a DNA polymerase template switching mechanism (Kent et al., 2016; van Kregten et al., 2016; Wyatt et al., 2016; Schimmel et al., 2017; Nishizawa-Yokoi et al., 2021).

Our analyses of hundreds of T-circles indicated that, collectively, all the properties of T-DNA/plant DNA junctions could be recapitulated by examining RB-LB junctions of T-circles. T-circles could be “complex,” resulting from linkage of multiple T-DNAs in either RB-LB configuration or in RB-RB/LB-LB configuration. T-DNA border deletions occur frequently, and are generally more extensive at the LB than at the RB. Use of the binary vector pE4636 indicated that deletions at the LB can be up to ~5 kbp (deletions at the RB > ~500 bp would not be tolerated in our T-circle recovery system because they would delete the *ColE1* origin of replication necessary to recover T-circles in *E. coli*). Filler DNA coming from various *Agrobacterium* and plant sources could occur between the RB and LB regions. Because T-circles are not integrated into plant DNA, the occurrence of plant DNA sequences in T-circles indicates that such plant DNA sequences are “copied” into the T-circles after arrival of T-strands in the nucleus. It is not clear how DNA from other *Agrobacterium* replicons links to T-circle sequences. Such linkage could occur in *Agrobacterium* prior to T-DNA transfer or could occur in the plant if these sequences were mobilized into the plant nucleus. Preliminary analysis of these *Agrobacterium* sequences indicates that they are not flanked by a consensus VirD2 cleavage site. Finally, microhomology frequently occurred between T-DNA (at or near the borders) and filler DNA, or the ends of deletions in T-DNA. Use of microhomology is an indication of a MMEJ process, perhaps using DNA polymerase θ.

### Use of *Agrobacterium* and Plant Proteins for T-Circle Formation

Although VirD2 protein remains attached to T-strands as they enter the nucleus, the role, if any, for VirD2 in T-DNA integration remains unknown. VirD2 is involved in many processes upstream of T-DNA integration, and mutations in VirD2 that decrease integration may also be impaired in one or more of these processes. Despite these potential complications, Tinland et al. (1995) showed that alteration of VirD2 arginine^129^ to glycine affected the precision of insertion of T-DNA into plant DNA; this mutation resulted in more deletions at integrated RBs. Mysore et al. (1998) showed that substitution of four serine residues for the VirD2 *ω* region sequence DDGR did not alter the general pattern of T-DNA integration, but preferentially decreased the extent of T-DNA integration (as measured by stable transformation) relative to transient transformation. Our studies on T-circles generated using an *Agrobacterium* strain with the *virD2 ω* mutation indicated that the frequency of T-circle formation was 2.7% that of T-circles formed using an *Agrobacterium* strain containing a wild-type *VirD2* gene. These data correlate well with past estimates of the decrease in T-DNA integration using this *virD2 ω* substitution mutant (Mysore et al., 1998). In addition, the precision of RBs (and to a lesser extent, LBs) in T-circles generated using the *virD2 ω* substitution mutant was different from those generated using a wild-type *VirD2* gene: All of the 23 T-circles examined from *N. benthamiana* using the *virD2 ω* substitution mutant contained precise RBs, whereas a lower percentage of T-circles derived using a wild-type *VirD2 Agrobacterium* strain contained precise RBs (81% using the initial T-circle binary vectors, 53% using the new T-circle binary vector pE4636). A lack of extensive RB deletions using the VirD2 *ω* mutant was previously noted (Mysore et al., 1998). Taken together, these data indicate that VirD2 protein, and especially the *ω* domain, influence the precision of both RBs and LBs in T-circles, and by extrapolation in T-DNA integration. The VirD2 *ω* mutant protein may remain on the T-strand longer than does wild-type VirD2 during T-circle formation, thus protecting it more extensively from nuclease degradation. Alternatively, the VirD2 *ω* mutant protein may block the activity of proteins involved in microhomology searching near the borders. Future experiments will examine these possibilities.

The role of Ku80 in T-DNA integration is controversial, as described above. Using a *ku80* mutant *Arabidopsis* line, we showed no decrease in the frequency of T-circle formation. Neither did we find any major differences among T-circles generated in wild-type vs. *ku80* mutant *Arabidopsis* plants with regard to their RB-LB junctions. These results are consistent with the model that Ku80, and therefore the cNHEJ pathway, is not essential for either T-circle formation or for T-DNA integration.

### T-Circles and T-DNA Integration

T-strands enter the plant nucleus as single-strand molecules (Tinland et al., 1994; Yusibov et al., 1994) that can be converted to double-strand linear or double-strand circular molecules. It is not known which of these three forms of T-DNA serve as the substrate, or replication template in the case of single-strand molecules, for integration. Double-strand circular T-DNA molecules have been isolated from *Agrobacterium*-infected yeast cells (Bundock et al., 1995; Rolloos et al., 2014). Examination of the border regions of these circles indicated that they were always precise, with nucleotides 1–3 of the RB linked to nucleotides 4–25 of the LB. Thus, studying T-circles isolated from yeast may not serve as a good model for T-DNA integration in plants.

Bakkeren et al. (1989) inserted a Cauliflower Mosaic Virus (CaMV) replicon in a T-DNA region of a binary vector. Infection of tobacco plants by *Agrobacterium* containing this binary vector resulted in circular CaMV replicons joined at or near the T-DNA borders. Analysis of these border region junctions indicated structures similar to what has been seen at T-DNA/plant DNA junctions: RBs were near-precise, and more extensive deletions occurred at the LB. Short “filler” DNA sequences between the border regions were seen in about one third of the molecules. These T-DNA border characteristics are similar to what we saw in our extensive T-circle analyses.

One peculiarity of our results was the different complexity of T-circles isolated from *N. benthamiana* and *Arabidopsis*. T-circle molecules isolated from *N. benthamiana* were frequently complex, with extensive DNA deletions, rearrangements, and filler DNA occurring at or near the border junctions. T-circles isolated from *Arabidopsis* were mostly simple T-DNA monomers with precise RB and LB junctions. It is not clear whether these differences reflect the disparate host plant species used or the different methods of *Agrobacterium* infection. Leaf infiltration of *Arabidopsis* remains very inefficient, although a recent protocol shows improvement of *Arabidopsis* leaf infiltration efficiency (Zhang et al., 2020). We are currently attempting to isolate T-circles from *Arabidopsis* leaves using a modification of this method.

Although the T-circle border junctions that we and others (Bakkeren et al., 1989) have examined closely resemble the range of border junction characteristics seen in integrated T-DNA molecules, we cannot argue that T-DNA circles are the substrate for integration into plant DNA. Rather, we propose that investigation of the mechanism of T-circle formation in plants may serve as a proxy for studying the events, and molecules, involved in T-DNA integration.

## Data Availability Statement

The datasets presented in this study can be found in the online repository NCBI GenBank under the accession numbers BankIt2568117 and BankIt2568514.

## Author Contributions

KS, L-YL, and SG designed the research. KS, L-YL, JY, and SG performed the research. KS, L-YL, and SG analyzed the data. KS and SG wrote the manuscript with input from L-YL and JY. All authors contributed to the article and approved the submitted version.

## Funding

This work was supported by a grant from the NSF (#1725122). We also wish to acknowledge support from the Purdue Center for Cancer Research *via* an NIH NCI grant (P30 CA023168), which supports the DNA Sequencing shared resources that were utilized in this work.

## Conflict of Interest

The authors declare that the research was conducted in the absence of any commercial or financial relationships that could be construed as a potential conflict of interest.

## Publisher’s Note

All claims expressed in this article are solely those of the authors and do not necessarily represent those of their affiliated organizations, or those of the publisher, the editors and the reviewers. Any product that may be evaluated in this article, or claim that may be made by its manufacturer, is not guaranteed or endorsed by the publisher.
