# Supplementary material for: Characterization of T-Circles and Their Formation Reveal Similarities to Agrobacterium T-DNA Integration Patterns
Source: Front Plant Sci. 2022 May 6;13:849930. doi: 10.3389/fpls.2022.849930 (PMC9121065; doi:10.3389/fpls.2022.849930)
Supplement: Supplementary file 1 [file Data_Sheet_1.pdf]

Supplemental Table 1. T-circles isolated in the initial experiments

No.	Sample No.	Strain	Construct	Monomeric/Complex*
N. benthamiana with TET-ORI:				
1	#001-1	EHA105	TET-ORI	complex
2	#001-2	EHA105	TET-ORI	monomeric
3	#001-4	EHA105	TET-ORI	monomeric
4	#001-5	EHA105	TET-ORI	monomeric
5	#001-6	EHA105	TET-ORI	monomeric
6	#002-19	EHA105	TET-ORI	monomeric
7	#002-20	EHA105	TET-ORI	complex
8	#002-21	EHA105	TET-ORI	complex
9	#002-22	EHA105	TET-ORI	monomeric
10	#002-23	EHA105	TET-ORI	complex
11	#002-25	EHA105	TET-ORI	monomeric
12	#002-26	EHA105	TET-ORI	monomeric
13	#003-50	EHA105	TET-ORI	complex
14	#003-51	EHA105	TET-ORI	complex
15	#003-52	EHA105	TET-ORI	monomeric
16	#003-53	EHA105	TET-ORI	monomeric
17	#003-54	EHA105	TET-ORI	complex
18	#003-55	EHA105	TET-ORI	monomeric
19	#003-56	EHA105	TET-ORI	monomeric
20	#003-57	EHA105	TET-ORI	monomeric
21	#003-58	EHA105	TET-ORI	monomeric
22	#003-59	EHA105	TET-ORI	monomeric
23	#003-60	EHA105	TET-ORI	complex
24	#003-61	EHA105	TET-ORI	complex
25	#003-62	EHA105	TET-ORI	complex
26	#003-63	EHA105	TET-ORI	monomeric
27	#003-64	EHA105	TET-ORI	complex
28	#003-65	EHA105	TET-ORI	monomeric
29	#003-66	EHA105	TET-ORI	monomeric
30	#003-67	EHA105	TET-ORI	monomeric
31	#003-68	EHA105	TET-ORI	complex
32	#003-69	EHA105	TET-ORI	monomeric
33	#003-70	EHA105	TET-ORI	monomeric
34	#008-49	EHA105	TET-ORI	complex
35	#008-50	EHA105	TET-ORI	complex
36	#008-51	EHA105	TET-ORI	complex
37	#008-52	EHA105	TET-ORI	monomeric
38	#008-53	EHA105	TET-ORI	complex
39	#008-54	EHA105	TET-ORI	monomeric
40	#008-55	EHA105	TET-ORI	monomeric
41	#008-57	EHA105	TET-ORI	monomeric
42	#008-58	EHA105	TET-ORI	monomeric
43	#008-59	EHA105	TET-ORI	monomeric
44	#008-60	EHA105	TET-ORI	complex
45	#008-61	EHA105	TET-ORI	complex

46	#008-62	EHA105	TET-ORI	complex
47	#008-63	EHA105	TET-ORI	complex
48	#008-64	EHA105	TET-ORI	monomeric
49	#008-65	EHA105	TET-ORI	monomeric
50	#008-66	EHA105	TET-ORI	complex
51	#008-67	EHA105	TET-ORI	monomeric
52	#008-68	EHA105	TET-ORI	monomeric
53	#008-69	EHA105	TET-ORI	monomeric
54	#008-70	EHA105	TET-ORI	monomeric
55	#008-71	EHA105	TET-ORI	monomeric
56	#008-72	EHA105	TET-ORI	monomeric
57	#008-73	EHA105	TET-ORI	complex
58	#008-74	EHA105	TET-ORI	monomeric
59	#008-75	EHA105	TET-ORI	monomeric
60	#008-76	EHA105	TET-ORI	complex
61	#008-77	EHA105	TET-ORI	complex
62	#008-78	EHA105	TET-ORI	monomeric
63	#008-79	EHA105	TET-ORI	complex
64	#008-80	EHA105	TET-ORI	complex
65	#008-81	EHA105	TET-ORI	monomeric
66	#008-82	EHA105	TET-ORI	monomeric
67	#008-83	EHA105	TET-ORI	complex
68	#008-84	EHA105	TET-ORI	complex
69	#008-85	EHA105	TET-ORI	complex
70	#008-86	EHA105	TET-ORI	complex
71	#008-87	EHA105	TET-ORI	monomeric
72	#008-88	EHA105	TET-ORI	complex
73	#008-89	EHA105	TET-ORI	monomeric
74	#008-90	EHA105	TET-ORI	complex
75	#009-1	EHA105	TET-ORI	complex
76	#009-2	EHA105	TET-ORI	monomeric
77	#009-3	EHA105	TET-ORI	monomeric
78	#009-4	EHA105	TET-ORI	monomeric
79	#009-5	EHA105	TET-ORI	complex
80	#009-10	EHA105	TET-ORI	complex
81	#009-11	EHA105	TET-ORI	monomeric
82	#009-12	EHA105	TET-ORI	complex
83	#009-13	EHA105	TET-ORI	monomeric
84	#009-14	EHA105	TET-ORI	complex
85	#009-15	EHA105	TET-ORI	monomeric
86	#009-16	EHA105	TET-ORI	monomeric
87	#009-17	EHA105	TET-ORI	monomeric
88	#009-18	EHA105	TET-ORI	monomeric
89	#009-19	EHA105	TET-ORI	monomeric
90	#009-21	EHA106	TET-ORI	monomeric
91	#009-22	EHA105	TET-ORI	complex
92	#009-23	EHA105	TET-ORI	monomeric
93	#009-24	EHA105	TET-ORI	monomeric

94	#009-25	EHA105	TET-ORI	monomeric
95	#009-26	EHA105	TET-ORI	monomeric
96	#009-27	EHA105	TET-ORI	monomeric
97	#009-28	EHA105	TET-ORI	monomeric
98	#011-29	EHA105	TET-ORI	complex
99	#011-30	EHA105	TET-ORI	monomeric
100	#011-31	EHA105	TET-ORI	complex
101	#011-32	EHA105	TET-ORI	complex
102	#011-33	EHA105	TET-ORI	complex
103	#011-34	EHA105	TET-ORI	monomeric
104	#011-35	EHA105	TET-ORI	monomeric
105	#011-36	EHA105	TET-ORI	monomeric
106	#011-37	EHA105	TET-ORI	monomeric
107	#011-38	EHA105	TET-ORI	monomeric

N. benthamiana with AMP-ORI:

1	#050-1	EHA105	AMP-ORI	monomeric
2	#050-2	EHA105	AMP-ORI	monomeric
3	#050-3	EHA105	AMP-ORI	monomeric
4	#050-4	EHA105	AMP-ORI	monomeric
5	#050-5	EHA105	AMP-ORI	monomeric
6	#050-6	EHA105	AMP-ORI	monomeric
7	#050-7	EHA105	AMP-ORI	complex
8	#050-8	EHA105	AMP-ORI	complex
9	#050-9	EHA105	AMP-ORI	monomeric
10	#050-10	EHA105	AMP-ORI	monomeric
11	#050-11	EHA105	AMP-ORI	monomeric
12	#050-12	EHA105	AMP-ORI	complex
13	#050-13	EHA105	AMP-ORI	complex
14	#050-14	EHA105	AMP-ORI	complex
15	#050-15	EHA105	AMP-ORI	monomeric
16	#050-16	EHA105	AMP-ORI	monomeric
17	#050-17	EHA105	AMP-ORI	complex
18	#050-18	EHA105	AMP-ORI	monomeric
19	#050-19	EHA105	AMP-ORI	monomeric
20	#050-21	EHA105	AMP-ORI	monomeric
21	#050-22	EHA105	AMP-ORI	complex
22	#050-23	EHA105	AMP-ORI	complex
23	#050-24	EHA105	AMP-ORI	monomeric
24	#050-25	EHA105	AMP-ORI	monomeric
25	#050-26	EHA105	AMP-ORI	monomeric
26	#050-27	EHA105	AMP-ORI	monomeric
27	#050-28	EHA105	AMP-ORI	monomeric
28	#050-29	EHA105	AMP-ORI	complex
29	#050-30	EHA105	AMP-ORI	monomeric
30	#050-31	EHA105	AMP-ORI	complex
31	#050-33	EHA105	AMP-ORI	monomeric
32	#050-34	EHA105	AMP-ORI	monomeric

33	#050-35	EHA105	AMP-ORI	monomeric
34	#050-36	EHA105	AMP-ORI	monomeric
35	#050-37	EHA105	AMP-ORI	monomeric
36	#050-38	EHA105	AMP-ORI	monomeric
37	#050-39	EHA105	AMP-ORI	monomeric
38	#050-40	EHA105	AMP-ORI	monomeric
39	#052-1	EHA105	AMP-ORI	complex
40	#052-2	EHA105	AMP-ORI	complex
41	#052-3	EHA105	AMP-ORI	monomeric
42	#052-4	EHA105	AMP-ORI	monomeric
43	#052-5	EHA105	AMP-ORI	monomeric
44	#052-6	EHA105	AMP-ORI	monomeric
45	#052-7	EHA105	AMP-ORI	monomeric
46	#052-8	EHA105	AMP-ORI	monomeric
47	#052-9	EHA105	AMP-ORI	complex
48	#052-10	EHA105	AMP-ORI	monomeric
49	#052-11	EHA105	AMP-ORI	monomeric
50	#052-12	EHA105	AMP-ORI	complex
51	#052-13	EHA105	AMP-ORI	monomeric
52	#052-14	EHA105	AMP-ORI	monomeric
53	#052-15	EHA105	AMP-ORI	monomeric
54	#052-16	EHA105	AMP-ORI	monomeric
55	#052-17	EHA105	AMP-ORI	complex
56	#052-18	EHA105	AMP-ORI	complex
57	#052-19	EHA105	AMP-ORI	monomeric
58	#052-20	EHA105	AMP-ORI	monomeric
59	#052-21	EHA105	AMP-ORI	complex
60	#052-22	EHA105	AMP-ORI	complex
61	#052-23	EHA105	AMP-ORI	monomeric
62	#052-24	EHA105	AMP-ORI	monomeric
63	#052-25	EHA105	AMP-ORI	complex
64	#052-26	EHA105	AMP-ORI	monomeric
65	#052-27	EHA105	AMP-ORI	complex
66	#052-28	EHA105	AMP-ORI	complex
67	#052-29	EHA105	AMP-ORI	monomeric
68	#052-30	EHA105	AMP-ORI	monomeric
69	#052-31	EHA105	AMP-ORI	monomeric
70	#052-32	EHA105	AMP-ORI	complex
71	#052-33	EHA105	AMP-ORI	monomeric
72	#052-34	EHA105	AMP-ORI	complex
73	#052-35	EHA105	AMP-ORI	complex
74	#052-36	EHA105	AMP-ORI	monomeric
75	#052-37	EHA105	AMP-ORI	monomeric
76	#052-38	EHA105	AMP-ORI	monomeric
77	#052-40	EHA105	AMP-ORI	monomeric
78	#052-41	EHA105	AMP-ORI	monomeric
79	#052-42	EHA105	AMP-ORI	complex
80	#052-43	EHA105	AMP-ORI	monomeric

81	#052-44	EHA105	AMP-ORI	complex
82	#052-45	EHA105	AMP-ORI	complex
83	#052-46	EHA105	AMP-ORI	monomeric
84	#052-48	EHA105	AMP-ORI	monomeric
85	#052-49	EHA105	AMP-ORI	monomeric
86	#052-50	EHA105	AMP-ORI	monomeric
87	#052-51	EHA105	AMP-ORI	monomeric
88	#052-52	EHA105	AMP-ORI	monomeric
89	#052-53	EHA105	AMP-ORI	complex
90	#052-54	EHA105	AMP-ORI	monomeric
91	#052-55	EHA105	AMP-ORI	complex
92	#052-56	EHA105	AMP-ORI	monomeric
93	#052-57	EHA105	AMP-ORI	monomeric
94	#052-58	EHA105	AMP-ORI	monomeric
95	#052-59	EHA105	AMP-ORI	monomeric
96	#052-60	EHA105	AMP-ORI	complex
97	#052-61	EHA105	AMP-ORI	monomeric
98	#052-62	EHA105	AMP-ORI	monomeric
99	#052-63	EHA105	AMP-ORI	complex
100	#052-64	EHA105	AMP-ORI	monomeric
101	#052-65	EHA105	AMP-ORI	monomeric
102	#052-66	EHA105	AMP-ORI	complex
103	#052-67	EHA105	AMP-ORI	monomeric
104	#052-68	EHA105	AMP-ORI	complex

N. benthamiana with *VirD2*Ω:

1	#005-1	At1959	TET-ORI	monomeric
2	#005-2	At1959	TET-ORI	monomeric
3	#005-3	At1959	TET-ORI	monomeric
4	#005-4	At1959	TET-ORI	complex
5	#005-6	At1959	TET-ORI	complex
6	#005-7	At1959	TET-ORI	monomeric
7	#005-8	At1959	TET-ORI	complex
8	#005-9	At1959	TET-ORI	monomeric
9	#055-1	At1959	AMP-ORI	monomeric
10	#055-2	At1959	AMP-ORI	monomeric

Arabidopsis Col-0

1	#021-1	EHA105	AMP-ORI	monomeric
2	#021-2	EHA105	AMP-ORI	monomeric
3	#021-3	EHA105	AMP-ORI	complex
4	#021-4	EHA105	AMP-ORI	complex
5	#021-5	EHA105	AMP-ORI	monomeric
6	#021-6	EHA105	AMP-ORI	monomeric
7	#021-7	EHA105	AMP-ORI	monomeric
8	#021-8	EHA105	AMP-ORI	monomeric
9	#021-9	EHA105	AMP-ORI	monomeric
10	#021-10	EHA105	AMP-ORI	monomeric
11	#021-11	EHA105	AMP-ORI	monomeric
12	#021-12	EHA105	AMP-ORI	monomeric

13	#021-13	EHA105	AMP-ORI	monomeric
Arabidopsis efr-1				
1	#022-1	EHA105	AMP-ORI	monomeric
2	#022-2	EHA105	AMP-ORI	monomeric
3	#022-3	EHA105	AMP-ORI	monomeric
4	#022-4	EHA105	AMP-ORI	monomeric
5	#022-5	EHA105	AMP-ORI	monomeric
6	#022-6	EHA105	AMP-ORI	monomeric
7	#022-7	EHA105	AMP-ORI	monomeric
8	#022-8	EHA105	AMP-ORI	monomeric
9	#022-9	EHA105	AMP-ORI	monomeric
10	#022-10	EHA105	AMP-ORI	monomeric
11	#022-11	EHA105	AMP-ORI	monomeric
12	#022-12	EHA105	AMP-ORI	monomeric
13	#022-13	EHA105	AMP-ORI	monomeric
14	#022-14	EHA105	AMP-ORI	monomeric
15	#022-15	EHA105	AMP-ORI	monomeric
16	#022-16	EHA105	AMP-ORI	monomeric
17	#022-17	EHA105	AMP-ORI	monomeric
18	#022-18	EHA105	AMP-ORI	monomeric
19	#022-19	EHA105	AMP-ORI	monomeric
20	#022-20	EHA105	AMP-ORI	monomeric
21	#022-21	EHA105	AMP-ORI	monomeric
22	#022-22	EHA105	AMP-ORI	monomeric
23	#022-23	EHA105	AMP-ORI	monomeric
24	#022-24	EHA105	AMP-ORI	monomeric
25	#022-25	EHA105	AMP-ORI	monomeric
26	#022-26	EHA105	AMP-ORI	monomeric
27	#022-27	EHA105	AMP-ORI	monomeric
28	#022-28	EHA105	AMP-ORI	monomeric
29	#022-29	EHA105	AMP-ORI	monomeric
30	#022-30	EHA105	AMP-ORI	monomeric
31	#022-31	EHA105	AMP-ORI	monomeric
32	#022-32	EHA105	AMP-ORI	monomeric
33	#022-33	EHA105	AMP-ORI	monomeric
34	#022-34	EHA105	AMP-ORI	monomeric
35	#022-35	EHA105	AMP-ORI	monomeric
36	#022-36	EHA105	AMP-ORI	monomeric
37	#022-37	EHA105	AMP-ORI	monomeric
38	#022-38	EHA105	AMP-ORI	monomeric
39	#022-39	EHA105	AMP-ORI	monomeric
40	#022-40	EHA105	AMP-ORI	monomeric
41	#022-41	EHA105	AMP-ORI	monomeric
42	#022-42	EHA105	AMP-ORI	monomeric
43	#022-43	EHA105	AMP-ORI	monomeric
44	#022-44	EHA105	AMP-ORI	monomeric
45	#022-45	EHA105	AMP-ORI	monomeric

46	#022-46	EHA105	AMP-ORI	monomeric
47	#022-47	EHA105	AMP-ORI	monomeric
48	#022-48	EHA105	AMP-ORI	monomeric
49	#025-49	EHA105	AMP-ORI	monomeric
50	#046-1	EHA105	AMP-ORI	monomeric
51	#046-2	EHA105	AMP-ORI	monomeric
52	#046-3	EHA105	AMP-ORI	monomeric
53	#046-4	EHA105	AMP-ORI	monomeric
54	#046-5	EHA105	AMP-ORI	monomeric
55	#046-6	EHA105	AMP-ORI	monomeric
56	#046-7	EHA105	AMP-ORI	monomeric
57	#046-8	EHA105	AMP-ORI	monomeric
58	#046-9	EHA105	AMP-ORI	monomeric
59	#046-10	EHA105	AMP-ORI	monomeric
60	#046-11	EHA105	AMP-ORI	monomeric
61	#046-12	EHA105	AMP-ORI	monomeric
62	#046-13	EHA105	AMP-ORI	monomeric
63	#046-14	EHA105	AMP-ORI	monomeric
64	#046-15	EHA105	AMP-ORI	monomeric
65	#046-16	EHA105	AMP-ORI	monomeric
66	#046-17	EHA105	AMP-ORI	monomeric
67	#046-18	EHA105	AMP-ORI	monomeric
68	#046-19	EHA105	AMP-ORI	monomeric
69	#046-20	EHA105	AMP-ORI	monomeric
70	#046-21	EHA105	AMP-ORI	complex
71	#046-22	EHA105	AMP-ORI	monomeric
72	#046-23	EHA105	AMP-ORI	monomeric
73	#046-24	EHA105	AMP-ORI	monomeric
74	#046-25	EHA105	AMP-ORI	monomeric
75	#046-26	EHA105	AMP-ORI	monomeric
76	#046-27	EHA105	AMP-ORI	monomeric
77	#046-28	EHA105	AMP-ORI	monomeric
78	#046-29	EHA105	AMP-ORI	monomeric
79	#046-30	EHA105	AMP-ORI	monomeric
80	#046-31	EHA105	AMP-ORI	monomeric
81	#046-32	EHA105	AMP-ORI	monomeric
82	#046-33	EHA105	AMP-ORI	monomeric
83	#046-34	EHA105	AMP-ORI	monomeric
84	#046-35	EHA105	AMP-ORI	monomeric
85	#046-36	EHA105	AMP-ORI	monomeric
86	#046-37	EHA105	AMP-ORI	monomeric
87	#046-38	EHA105	AMP-ORI	monomeric
88	#046-39	EHA105	AMP-ORI	monomeric
89	#046-40	EHA105	AMP-ORI	monomeric
90	#046-41	EHA105	AMP-ORI	monomeric
91	#046-42	EHA105	AMP-ORI	monomeric
92	#046-43	EHA105	AMP-ORI	monomeric
93	#046-44	EHA105	AMP-ORI	monomeric

94	#046-45	EHA105	AMP-ORI	monomeric
95	#046-46	EHA105	AMP-ORI	monomeric
96	#046-47	EHA105	AMP-ORI	monomeric
97	#046-48	EHA105	AMP-ORI	monomeric
98	#046-49	EHA105	AMP-ORI	monomeric
99	#047-1	EHA105	AMP-ORI	monomeric
100	#047-2	EHA105	AMP-ORI	monomeric
101	#047-3	EHA105	AMP-ORI	complex
102	#047-4	EHA105	AMP-ORI	monomeric
103	#047-5	EHA105	AMP-ORI	monomeric
104	#047-6	EHA105	AMP-ORI	monomeric
105	#047-7	EHA105	AMP-ORI	monomeric
106	#047-8	EHA105	AMP-ORI	monomeric
107	#047-9	EHA105	AMP-ORI	monomeric
108	#047-11	EHA105	AMP-ORI	monomeric
109	#047-12	EHA105	AMP-ORI	monomeric
110	#047-13	EHA105	AMP-ORI	monomeric
111	#047-14	EHA105	AMP-ORI	monomeric
112	#047-15	EHA105	AMP-ORI	monomeric
113	#047-16	EHA105	AMP-ORI	monomeric
114	#047-17	EHA105	AMP-ORI	monomeric
115	#047-18	EHA105	AMP-ORI	monomeric
116	#047-19	EHA105	AMP-ORI	monomeric
117	#047-20	EHA105	AMP-ORI	monomeric
118	#047-21	EHA105	AMP-ORI	monomeric
119	#047-22	EHA105	AMP-ORI	monomeric
120	#047-23	EHA105	AMP-ORI	monomeric
121	#041-1	EHA105	AMP-ORI	monomeric
122	#041-2	EHA105	AMP-ORI	monomeric
123	#041-3	EHA105	AMP-ORI	monomeric
124	#041-4	EHA105	AMP-ORI	monomeric
125	#041-5	EHA105	AMP-ORI	monomeric
126	#041-6	EHA105	AMP-ORI	monomeric
127	#041-7	EHA105	AMP-ORI	monomeric
128	#041-8	EHA105	AMP-ORI	monomeric
129	#041-9	EHA105	AMP-ORI	monomeric
130	#041-10	EHA105	AMP-ORI	monomeric

Arabidopsis efr-1/ku80

1	#043-1	EHA105	AMP-ORI	monomeric
2	#043-2	EHA105	AMP-ORI	monomeric
3	#043-3	EHA105	AMP-ORI	monomeric
4	#043-4	EHA105	AMP-ORI	monomeric
5	#043-5	EHA105	AMP-ORI	monomeric
6	#043-6	EHA105	AMP-ORI	monomeric
7	#043-7	EHA105	AMP-ORI	monomeric
8	#043-8	EHA105	AMP-ORI	monomeric
9	#043-9	EHA105	AMP-ORI	monomeric

10	#043-10	EHA105	AMP-ORI	monomeric
11	#043-11	EHA105	AMP-ORI	monomeric
12	#043-12	EHA105	AMP-ORI	monomeric
13	#043-15	EHA105	AMP-ORI	monomeric
14	#043-16	EHA105	AMP-ORI	monomeric
15	#043-17	EHA105	AMP-ORI	monomeric
16	#043-18	EHA105	AMP-ORI	monomeric
17	#043-19	EHA105	AMP-ORI	monomeric
18	#043-20	EHA105	AMP-ORI	monomeric
19	#043-21	EHA105	AMP-ORI	monomeric
20	#043-22	EHA105	AMP-ORI	monomeric
21	#043-23	EHA105	AMP-ORI	monomeric
22	#043-24	EHA105	AMP-ORI	monomeric
23	#043-25	EHA105	AMP-ORI	complex
24	#043-26	EHA105	AMP-ORI	monomeric
25	#043-27	EHA105	AMP-ORI	monomeric
26	#043-28	EHA105	AMP-ORI	monomeric
27	#043-29	EHA105	AMP-ORI	monomeric
28	#043-30	EHA105	AMP-ORI	complex
29	#043-31	EHA105	AMP-ORI	monomeric
30	#043-32	EHA105	AMP-ORI	monomeric
31	#043-33	EHA105	AMP-ORI	monomeric
32	#043-34	EHA105	AMP-ORI	monomeric
33	#043-35	EHA105	AMP-ORI	monomeric
34	#043-36	EHA105	AMP-ORI	monomeric
35	#043-37	EHA105	AMP-ORI	monomeric
36	#043-38	EHA105	AMP-ORI	monomeric
37	#043-39	EHA105	AMP-ORI	monomeric
38	#043-40	EHA105	AMP-ORI	monomeric
39	#043-41	EHA105	AMP-ORI	complex
40	#043-42	EHA105	AMP-ORI	monomeric
41	#043-43	EHA105	AMP-ORI	monomeric
42	#043-44	EHA105	AMP-ORI	monomeric
43	#043-45	EHA105	AMP-ORI	monomeric
44	#043-46	EHA105	AMP-ORI	monomeric
45	#043-47	EHA105	AMP-ORI	monomeric
46	#045-1	EHA105	AMP-ORI	monomeric
47	#045-2	EHA105	AMP-ORI	monomeric
48	#045-3	EHA105	AMP-ORI	monomeric
49	#045-4	EHA105	AMP-ORI	monomeric
50	#045-5	EHA105	AMP-ORI	monomeric
51	#045-6	EHA105	AMP-ORI	monomeric
52	#045-7	EHA105	AMP-ORI	monomeric
53	#045-8	EHA105	AMP-ORI	monomeric
54	#045-9	EHA105	AMP-ORI	monomeric
55	#045-10	EHA105	AMP-ORI	monomeric
56	#045-11	EHA105	AMP-ORI	monomeric
57	#045-12	EHA105	AMP-ORI	monomeric

58	#045-13	EHA105	AMP-ORI	complex
59	#045-14	EHA105	AMP-ORI	monomeric
60	#045-15	EHA105	AMP-ORI	monomeric
61	#045-16	EHA105	AMP-ORI	monomeric
62	#045-17	EHA105	AMP-ORI	monomeric
63	#045-18	EHA105	AMP-ORI	monomeric
64	#045-19	EHA105	AMP-ORI	monomeric
65	#045-20	EHA105	AMP-ORI	monomeric
66	#045-21	EHA105	AMP-ORI	monomeric
67	#045-22	EHA105	AMP-ORI	monomeric
68	#045-23	EHA105	AMP-ORI	monomeric
69	#045-24	EHA105	AMP-ORI	monomeric
70	#045-25	EHA105	AMP-ORI	monomeric
71	#045-26	EHA105	AMP-ORI	monomeric
72	#045-27	EHA105	AMP-ORI	monomeric
73	#045-28	EHA105	AMP-ORI	monomeric
74	#045-29	EHA105	AMP-ORI	monomeric
75	#045-30	EHA105	AMP-ORI	monomeric

* Determination of monomeric or complex structure was done by agarose gel analysis. When result was uncertain T-circles were further sequenced and classified accordingly.

Supplemental Table 2. Sequenced T-DNA junctions of monomeric T-circles from *N. benthamiana*

Sample No.	Strain	Construct	RB[a]	Microhomology	Filler DNA	LB[a]
#001-2	EHA105	TET-ORI	precise	0	1 (T)	-25
#001-4	EHA105	TET-ORI	precise	0	0	-11
#001-5	EHA105	TET-ORI	-2	2 (TT)	0	-32
#001-6	EHA105	TET-ORI	precise	12[b]	0	precise
#002-19	EHA105	TET-ORI	precise	12[b]	0	precise
#002-22	EHA105	TET-ORI	precise	12[b]	0	precise
#002-25	EHA105	TET-ORI	precise	0	0	-82
#002-26	EHA105	TET-ORI	precise	12[b]	0	precise
#003-52	EHA105	TET-ORI	precise	12[b]	0	precise
#003-53	EHA105	TET-ORI	precise	12[b]	0	precise
#003-55	EHA105	TET-ORI	precise + 2[c]	2 (CA)	0	-98
#003-56	EHA105	TET-ORI	precise	0	1 (T)	-23
#003-57	EHA105	TET-ORI	precise	12[b]	0	precise
#003-58	EHA105	TET-ORI	-1	3 (TTG)	0	-52
#003-59	EHA105	TET-ORI	precise	12[b]	0	precise
#003-63	EHA105	TET-ORI	precise	12[b]	0	precise
#003-65	EHA105	TET-ORI	precise	0	0	-65
#003-66	EHA105	TET-ORI	precise	12[b]	0	precise
#003-67	EHA105	TET-ORI	precise	12[b]	0	precise
#003-70	EHA105	TET-ORI	precise	12[b]	0	precise
#008-55	EHA105	TET-ORI	precise	12[b]	0	precise
#008-57	EHA105	TET-ORI	-2	0	1 (A)	-27
#008-59	EHA105	TET-ORI	precise	12[b]	0	precise
#008-67	EHA105	TET-ORI	precise	12[b]	0	precise
#008-68	EHA105	TET-ORI	precise	12[b]	0	precise
#008-69	EHA105	TET-ORI	precise + 2 bp[c]	2 (CA)	0	-1
#008-71	EHA105	TET-ORI	precise	12[b]	0	precise
#008-72	EHA105	TET-ORI	precise	0	1 (A)	-69
#008-75	EHA105	TET-ORI	precise	0	1 (A)	-8
#008-82	EHA105	TET-ORI	precise	12[b]	0	precise
#009-2	EHA105	TET-ORI	-3	0	0	-71
#009-3	EHA105	TET-ORI	precise	0	4 (AAAA)	-175
#009-4	EHA105	TET-ORI	precise	12[b]	0	precise
#009-11	EHA105	TET-ORI	precise	12[b]	0	precise
#009-13	EHA105	TET-ORI	precise	0	0	-22
#009-17	EHA105	TET-ORI	precise	12[b]	0	precise
#009-18	EHA105	TET-ORI	precise	12[b]	0	precise
#009-19	EHA105	TET-ORI	precise	12[b]	0	precise

#009-21	EHA106	TET-ORI	precise	0	1 (T)	-96
#009-26	EHA105	TET-ORI	precise	0	0	-70
#011-30	EHA105	TET-ORI	-16	0	0	-16
#011-34	EHA105	TET-ORI	precise	12[b]	0	precise
#011-35	EHA105	TET-ORI	precise + 7 bp	10 (TGTTTGACAG)	0	-100
#011-36	EHA105	TET-ORI	precise	0	0	-62
#011-38	EHA105	TET-ORI	precise	12[b]	0	precise
#050-1	EHA105	AMP-ORI	precise	12	0	precise
#050-5	EHA105	AMP-ORI	precise	0	1 (A)	-61
#050-6	EHA105	AMP-ORI	-1	1 (G)	0	-632
#050-9	EHA105	AMP-ORI	precise	12[b]	0	precise
#050-10	EHA105	AMP-ORI	precise	12[b]	0	precise
#050-11	EHA105	AMP-ORI	precise	0	0	-629
#050-18	EHA105	AMP-ORI	-291	0	2 (GT)	-264
#050-24	EHA105	AMP-ORI	-10	0	0	-229
#050-27	EHA105	AMP-ORI	precise	12[b]	0	precise
#050-30	EHA105	AMP-ORI	precise +[d]	1 (C)	0	-311
#050-35	EHA105	AMP-ORI	precise	12[b]	0	precise
#050-37	EHA105	AMP-ORI	-75	0	4 (AGCT)	-464
#050-40	EHA105	AMP-ORI	precise	0	0	-130
#052-4	EHA105	AMP-ORI	-39	0	0	-644
#052-5	EHA105	AMP-ORI	precise	0	1 (T)	-92
#052-13	EHA105	AMP-ORI	precise	12[b]	0	precise
#052-14	EHA105	AMP-ORI	precsie	12[b]	0	precise
#052-16	EHA105	AMP-ORI	precise	0	1 (A)	-761
#052-19	EHA105	AMP-ORI	-1	0	0	-408
#052-24	EHA105	AMP-ORI	precise +[d]	1 (C)	13 bp of T-DNA or binary vector sequence	-560
#052-26	EHA105	AMP-ORI	-2	0	0	-75
#052-29	EHA105	AMP-ORI	-24	0	0	-410
#052-30	EHA105	AMP-ORI	precise	0	0	-579
#052-38	EHA105	AMP-ORI	precise	12[b]	0	precise
#052-43	EHA105	AMP-ORI	precise	12[b]	0	precise
#052-46	EHA105	AMP-ORI	precise	1 (A)	0	-43
#052-47	EHA105	AMP-ORI	precise	0	1 (A)	-743
#052-48	EHA105	AMP-ORI	precise	12[b]	0	precise
#052-49	EHA105	AMP-ORI	precise	0	0	-758
#052-50	EHA105	AMP-ORI	precise	12[b]	0	precise
#052-51	EHA105	AMP-ORI	precise	12[b]	0	precise
#052-54	EHA105	AMP-ORI	-342	0	1 (G)	-536

#052-58	EHA105	AMP-ORI	precise	2 (GA)	0	-234
#052-62	EHA105	AMP-ORI	precise	12[b]	0	precise
#052-65	EHA105	AMP-ORI	-2	0	1 (A)	-25

[a]Right border (RB) and left border (LB) numerical values represent the position in DNA relative to precise end; [b]12 bp can be a readthrough sequence of RB involved in microhomology with LB, or a precise RB joined to a precise LB end (i.e., no readthrough and microhomology); [c]The two nucleotides after the precise RB (CA) can be a readthrough of a RB sequence involved in microhomology with a LB side, or precise RB without readthrough (CA comes from LB); [d]One nucleotide after precise RB (C) can be a readthrough of RB sequence involved in microhomology with LB side, or precise RB without readthrough (C comes from LB sequence).

Supplemental Table 3. Sequenced T-DNA junctions of monomeric T-circles from *Arabidopsis efr-1* and Col-0 plants

Background	Sample No.	Strain	Construct	RB[a]	Microhomology	Filler DNA	LB[a]
efr-1							
	#022-2	EHA105	AMP-ORI	precise	12[b]	0	precise
	#022-3	EHA105	AMP-ORI	precise	1 (A)	0	-22
	#022-4	EHA105	AMP-ORI	precise	12[b]	0	precise
	#022-5	EHA105	AMP-ORI	precise	12[b]	0	precise
	#022-6	EHA105	AMP-ORI	precise	12[b]	0	precise
	#022-7	EHA105	AMP-ORI	precise	0	5 (TAATA)	precise
	#022-8	EHA105	AMP-ORI	-8	0	26 (TTAAT AGTTTAA ACTGAAG CGCAGAT)	precise
	#022-9	EHA105	AMP-ORI	precise	12[b]	0	precise
	#022-10	EHA105	AMP-ORI	precise	12[b]	0	precise
	#022-11	EHA105	AMP-ORI	precise	12[b]	0	precise
	#022-12	EHA105	AMP-ORI	precise	12[b]	0	precise
	#022-13	EHA105	AMP-ORI	precise	12[b]	0	precise
	#022-14	EHA105	AMP-ORI	precise	12[b]	0	precise
	#022-15	EHA105	AMP-ORI	precise	12[b]	0	precise
	#022-16	EHA105	AMP-ORI	precise	12[b]	0	precise
	#022-17	EHA105	AMP-ORI	precise	12[b]	0	precise
	#022-18	EHA105	AMP-ORI	precise	0	1 (T)	-9
	#022-19	EHA105	AMP-ORI	precise	12[b]	0	precise
	#022-20	EHA105	AMP-ORI	precise	12[b]	0	precise
	#022-22	EHA105	AMP-ORI	precise	12[b]	0	precise
	#022-23	EHA105	AMP-ORI	precise	12[b]	0	precise
	#022-24	EHA105	AMP-ORI	precise	12[b]	0	precise
	#022-25	EHA105	AMP-ORI	precise	12[b]	0	precise
	#022-26	EHA105	AMP-ORI	-1	1 (G)	0	-16
	#022-27	EHA105	AMP-ORI	precise	0	1 (A)	-15
	#022-28	EHA105	AMP-ORI	precise	12[b]	0	precise
	#022-29	EHA105	AMP-ORI	precise	12[b]	0	precise
	#022-30	EHA105	AMP-ORI	precise	12[b]	0	precise
	#022-31	EHA105	AMP-ORI	precise	12[b]	0	precise
	#022-32	EHA105	AMP-ORI	precise	12[b]	0	precise
	#022-33	EHA105	AMP-ORI	precise	12[b]	0	precise
	#022-34	EHA105	AMP-ORI	-1	3 (TTG)	0	-9
	#022-35	EHA105	AMP-ORI	precise	12[b]	0	precise
	#022-36	EHA105	AMP-ORI	precise	12[b]	0	precise
	#022-37	EHA105	AMP-ORI	-13	4 (AAAC)	0	-18

#022-38	EHA105	AMP-ORI	precise	12[b]	0	precise
#022-39	EHA105	AMP-ORI	precise	12[b]	0	precise
#022-40	EHA105	AMP-ORI	precise	12[b]	0	precise
#022-41	EHA105	AMP-ORI	precise	1 (A)	0	-6
#022-42	EHA105	AMP-ORI	precise	12[b]	0	precise
#022-43	EHA105	AMP-ORI	precise	12[b]	0	precise
#022-44	EHA105	AMP-ORI	precise	12[b]	0	precise
#022-45	EHA105	AMP-ORI	precise	12[b]	0	precise
#022-46	EHA105	AMP-ORI	precise	12[b]	0	precise
#022-47	EHA105	AMP-ORI	precise	12[b]	0	precise
#022-48	EHA105	AMP-ORI	precise	12[b]	0	precise
#025-49	EHA105	AMP-ORI	precise	12[b]	0	precise
#046-1	EHA105	AMP-ORI	precise	12[b]	0	precise
#046-3	EHA105	AMP-ORI	precise	12[b]	0	precise
#046-4	EHA105	AMP-ORI	precise	12[b]	0	precise
#046-5	EHA105	AMP-ORI	precise	12[b]	0	precise
#046-6	EHA105	AMP-ORI	precise	12[b]	0	precise
#046-7	EHA105	AMP-ORI	precise	12[b]	0	precise
#046-10	EHA105	AMP-ORI	precise	12[b]	0	precise
#046-11	EHA105	AMP-ORI	precise	12[b]	0	precise
#046-12	EHA105	AMP-ORI	precise	12[b]	0	precise
#046-13	EHA105	AMP-ORI	precise	12[b]	0	precise
#046-14	EHA105	AMP-ORI	precise	12[b]	0	precise
#046-15	EHA105	AMP-ORI	precise	12[b]	0	precise
#046-16	EHA105	AMP-ORI	precise	12[b]	0	precise
#046-17	EHA105	AMP-ORI	precise	12[b]	0	precise
#046-18	EHA105	AMP-ORI	precise	12[b]	0	precise
#046-19	EHA105	AMP-ORI	precise	12[b]	0	precise
#046-20	EHA105	AMP-ORI	precise	12[b]	0	precise
#046-22	EHA105	AMP-ORI	precise	12[b]	0	precise
#046-23	EHA105	AMP-ORI	precise	12[b]	0	precise
#046-24	EHA105	AMP-ORI	precise	12[b]	0	precise
#046-26	EHA105	AMP-ORI	precise	12[b]	0	precise
#046-27	EHA105	AMP-ORI	precise	12[b]	0	precise
#046-28	EHA105	AMP-ORI	precise	12[b]	0	precise
#046-29	EHA105	AMP-ORI	precise	12[b]	0	precise
#046-30	EHA105	AMP-ORI	precise	12[b]	0	precise
#046-31	EHA105	AMP-ORI	precise	12[b]	0	precise
#046-32	EHA105	AMP-ORI	precise	12[b]	0	precise
#046-35	EHA105	AMP-ORI	precise	12[b]	0	precise

#046-36	EHA105	AMP-ORI	precise	12[b]	0	precise
#046-37	EHA105	AMP-ORI	-291	0	3 (GTC)	-15
#046-42	EHA105	AMP-ORI	precise	12[b]	0	precise
#046-43	EHA105	AMP-ORI	precise	12[b]	0	precise
#046-44	EHA105	AMP-ORI	precise	12[b]	0	precise
#046-45	EHA105	AMP-ORI	precise	12[b]	0	precise
#046-46	EHA105	AMP-ORI	precise	12[b]	0	precise
#046-47	EHA105	AMP-ORI	precise	12[b]	0	precise
#046-48	EHA105	AMP-ORI	precise	12[b]	0	precise
#046-49	EHA105	AMP-ORI	precise	12[b]	0	precise
#047-1	EHA105	AMP-ORI	precise	12[b]	0	precise
#047-2	EHA105	AMP-ORI	precise	12[b]	0	precise
#047-4	EHA105	AMP-ORI	precise	12[b]	0	precise
#047-5	EHA105	AMP-ORI	precise	12[b]	0	precise
#047-6	EHA105	AMP-ORI	precise	12[b]	0	precise
#047-7	EHA105	AMP-ORI	precise	12[b]	0	precise
#047-8	EHA105	AMP-ORI	precise	0	0	-581
#047-11	EHA105	AMP-ORI	precise	12[b]	0	precise
#047-13	EHA105	AMP-ORI	precise	12[b]	0	precise
#047-14	EHA105	AMP-ORI	precise	12[b]	0	precise
#047-15	EHA105	AMP-ORI	precise	12[b]	0	precise
#047-16	EHA105	AMP-ORI	precise	12[b]	0	precise
#047-17	EHA105	AMP-ORI	precise	12[b]	0	precise
#047-18	EHA105	AMP-ORI	precise	12[b]	0	precise
#047-19	EHA105	AMP-ORI	precise	12[b]	0	precise
#047-20	EHA105	AMP-ORI	precise	12[b]	0	precise
#047-21	EHA105	AMP-ORI	precise	12[b]	0	precise
#047-22	EHA105	AMP-ORI	precise	12[b]	0	precise
#047-23	EHA105	AMP-ORI	precise	12[b]	0	precise
#041-1	EHA105	AMP-ORI	precise	12[b]	0	precise
#041-2	EHA105	AMP-ORI	precise	12[b]	0	precise
#041-3	EHA105	AMP-ORI	precise	12[b]	0	precise
#041-5	EHA105	AMP-ORI	precise	12[b]	0	precise
#041-8	EHA105	AMP-ORI	precise	12[b]	0	precise
#041-9	EHA105	AMP-ORI	precise	12[b]	0	precise
#041-10	EHA105	AMP-ORI	precise	12[b]	0	precise

Col-0							
	#021-1	EHA105	AMP-ORI	precise	12[b]	0	precise
	#021-2	EHA105	AMP-ORI	precise	12[b]	0	precise
	#021-5	EHA105	AMP-ORI	precise	12[b]	0	precise
	#021-7	EHA105	AMP-ORI	precise	12[b]	0	precise
	#021-8	EHA105	AMP-ORI	precise	12[b]	0	precise
	#021-9	EHA105	AMP-ORI	precise	12[b]	0	precise
	#021-10	EHA105	AMP-ORI	precise	12[b]	0	precise
	#021-11	EHA105	AMP-ORI	precise	12[b]	0	precise
	#021-12	EHA105	AMP-ORI	precise	12[b]	0	precise
	#021-13	EHA105	AMP-ORI	precise	12[b]	0	precise

[a]Right border (RB) and left border (LB) numerical values represent the position in DNA relative to precise end; [b]Microhomology of 12 bp nucleotides is true only of there were a readthrough of a RB sequence. Alternately, a precise LB and precise LB were joined without microhomology.

Supplemental Table 4. Sequenced T-DNA junctions from heterodimeric KAN-ORI and TET-ORI T-circles

Sample No.	Constructs	RB-RB junction				LB-LB junction			
		RB (TET-ORI)	Microhomology	Filler DNA	RB (KAN-ORI)	LB (TET-ORI)	Microhomology	Filler DNA	LB (KAN-ORI)
#3	KAN-ORI and TET-ORI	-1	2 (CA)	0	precise	-105	0	1 (A)	-86
#4	KAN-ORI and TET-ORI	-231	2 (GA)	0	precise	-82	6 (TTCGGC)	0	-212
#5	KAN-ORI and TET-ORI	precise	0	0	precise	-98	3 (CAT)	0	-117
#6	KAN-ORI and TET-ORI	precise	1 (T)		-2	-81	2 (CG)	0	-29
#9	KAN-ORI and TET-ORI	precise	0	0	precise	-77	4 (TTAA)	0	-77
#10	KAN-ORI and TET-ORI	precise +2	2 (CA)	0	-1	-47	4 (ACAC)	0	-14
#11	KAN-ORI and TET-ORI	precise	0	3 (ATA)	precise	-66	5 (AATGT)	0	-49
#15	KAN-ORI and TET-ORI	precise	0	0	precise	-41	4 (TTAA)	0	-41
#16	KAN-ORI and TET-ORI	precise	0	0	precise	-83	1(T)	9 bp T-DNA sequence	-20
#18	KAN-ORI and TET-ORI	-12	1 (A)	0	precise	NA	NA	NA	NA
#22	KAN-ORI and TET-ORI	precise	0	0	precise	-100	4 (TGTT)	0	-46
#24	KAN-ORI and TET-ORI	precise +2	2 (CA)	0	-1	-100	4 (AACA)	0	-1018
#25	KAN-ORI and TET-ORI	precise +2	2 (CA)	0	-1	-25	1 (T)	0	-86
#30	KAN-ORI and TET-ORI	precise	0	0	precise	NA	NA	NA	NA
#32	KAN-ORI and TET-ORI	precise	0	0	precise	-26	2 (TG)	0	-264
#37	KAN-ORI and TET-ORI	precise	0	0	-2	-78	4 (GTTA)	0	-1061
		RB-LB junction				LB-RB junction			
		LB (TET-ORI)	Microhomology	Filler DNA	RB (KAN-ORI)	RB (TET-ORI)	Microhomology	Filler DNA	LB (KAN-ORI)
#17	KAN-ORI and TET-ORI	precise	12**	0	precise	precise	12**	0	precise
		LB-RB junction				RB-RB junction			
		LB (TET-ORI)	Microhomology	Filler DNA	RB (KAN-ORI)	RB (TET-ORI)	Microhomology	Filler DNA	RB (KAN-ORI)
#12*	KAN-ORI and TET-ORI	precise	12**	0	precise	precise	0	1 (A)	precise

*The third junction (between two T-DNA LB sides) in T-circle #12 was not sequenced; **12 bp can be a readthrough sequence of RB involved in microhomology with LB, or a precise RB joined to a precise LB end (i.e., no readthrough and microhomology)

Supplemental Table 5. Characterization of T-circles isolated from *Nicotiana benthamiana* using the T-DNA binary vector pE4636

T-circle ID	Sequencing Method	RB status	LB status	Microhomology at RB	Microhomology at LB	Filler DNA	Major rearrangements	Size (bp)[a,b]
M-1	Sanger	Precise	23 bp deletion	3 bp, but not directly at the border (TTGx)	3 bp (AAT)	303 bp from *N. benthamiana*	None	7814
K-10	Wide-Seq	327 bp deletion	4943 bp deletion	5 bp (GCGCC)	None	None	None	2241
J-1	Wide-Seq	Precise	89 bp deletion	None	None	None	None	6364
J-5	Wide-Seq	1 bp deletion	2072 bp deletion	None	None	1506 from pAtC58	None	6606
JYWT6	Sanger	Precise	?	None	None	None	From 97 to 1052 matched with expected T- circle 3720-2764 in reverse complement orientation	~ 7000
JYWT7	Sanger	Precise	484 bp deletion	None	None	736 bp from pAtC58	None	7674
JYWT8	Sanger	1 bp deletion	4352 bp deletion	2 bp	None	None	None	~3000
JYWT9	Sanger	7 bp deletion	4554 bp deletion	None	None	None	None	~3000
JYWT11	Sanger	1 bp deletion	4981 bp deletion	None	None	None	None	~2500
JY4	Sanger	Precise	?	None	None	801 bp from pE4636	Sequence ends with 801 bp from pE4636; needs Wide-Seq analysis	~12,000
JY6	Sanger	Precise	5972 bp deletion	None	None	None	The *bla* gene promoter and much of the *bla* gene are deleted, but there must be another full copy	~4000
4	Sanger	22 bp deletion	None	None	None	None	Inverted fragment insertion	~7500
10	WideSeq	327 bp deletion	4944 bp deletion	None	None	None	None	2241
4-3	Sanger	Precise	74 bp deletion	None	None	None	None	7437
4-10	Sanger	Precise	361 bp deletion	None	None	1 bp (T)	None	7151
4-7	Sanger	4 bp deletion	4610 bp deletion	None	None	None	None	2897
4-8	Sanger	83 bp deletion	3831 bp deletion	None	None	None	None	3622
4-12	Wide-Seq	456 bp deletion	4790 bp deletion	None	None	2667 bp from *N. benthamiana*	None	4932
4-14	Wide-Seq	458 bp deletion	4495 bp deletion	1 bp (T)	4 bp (ATCT)	None	1548 bp region from *Venus-intron* gene in inverted orientation	4106
4-23	Wide-Seq	362 bp deletion	4959 bp deletion	None	1 (T)	None	1328 bp from the *hptII* gene in inverted orientation	3520

4-20	Sanger	Precise	4816 bp deletion	None	None	None	None	2695
5-1	Sanger	3 bp deletion	4852 bp deletion	None	None	None	None	2656
5-2	Sanger	Precise	4801 bp deletion	None	None	None	None	2710
5-5	Sanger	Precise	88 bp deletion	2 bp (GA)	None	None	None	7424
6-2	Wide-Seq	Precise	4521 bp deletion	None	2 bp (TC)	1287 bp from the *hptII* gene, with part of the teminator	Inverted fragment insertion	4271
6-5	Sanger	Precise	5203 bp deletion	None	None	None	None	2308
6-6	WideSeq	Precise	4753 bp deletion	2 bp (GA)	None	3698 bp fragment from Venus cassette	inverted fragment insertion	6457
6-7	Sanger	Precise	5162 bp deletion	None	None	None	None	2349
6-8	WideSeq	Precise	4553 bp deletion	None	5 bp (AATGA)	1177 bp insertion (35S terminator)	Inverted fragment insertion	4263
6-9	Sanger	2 bp deletion	4291 bp deletion	None	None	2 bp (TG)	None	3220
6-15	Sanger	Precise	4622 bp deletion	2 bp (GA)	None	1 bp (T)	None	2890
6-19	Sanger	12 bp deletion	512 bp deletion	TTC vs. TTT	None	None	None	6978
6-24	Sanger	8 bp deletion	5326 bp deletion	None	None	1 bp (A) at the RB junction and 2 bp (AA) at the LB junction	Inverted fragment insertion	2889
6-28	Sanger	Precise	4842 bp deletion	None	2 bp (AA)	None	Inverted fragment insertion	2879
6-36	Sanger	Precise	220 bp deletion	None	None	None	None	7291
6-38	Sanger	322 bp deletion	4948 bp deletion	5 bp (GCGCC)	None	None	None	2241
6-39	Wide-Seq	Precise	4698 bp deletion	None	None	554 bp from pAtC58	Inverted fragment insertion	3368
6-41-21	Wide-Seq	1 bp deletion	2183 bp deletion	1 bp (G)	None	None	Inverted fragment from the binary vector	5964
6-42	Sanger	Precise	4222 bp deletion	1 bp (A)	None	None	None	3291
6-43-27	Sanger	18 bp deletion	4979 bp deletion	None	None	None	Inverted fragment (44 bp) insertion	2559
6-44	Sanger	138 bp deletion	446 bp deletion	4 bp (ATAA)	None	None	None	6927
6-51	Sanger	Precise	4990 bp deletion	2 out of 3 (TGA vs. TGC)	2 bp (AC)	None	Inverted fragment (290 bp) insertion	2811

[a]Sanger sequencing sizes are estimates based on the deletion sizes at the RB and LB

[b]Approximate sizes are based on restriction endonuclease fragment sizes

Supplemental Table 6. Sequenced T-DNA junctions of monomeric T-circles from *Arabidopsis ku80/efr-1* mutants

Sample No.	Strain	Construct	RB[a]	Microhomology	Filler DNA	LB[a]
#043-1	EHA105	AMP-ORI	Precise+2	4 (GACA)	0	-35
#043-2	EHA105	AMP-ORI	precise	12[b]	0	precise
#043-3	EHA105	AMP-ORI	precise	12[b]	0	precise
#043-4	EHA105	AMP-ORI	precise	12[b]	0	precise
#043-5	EHA105	AMP-ORI	precise	12[b]	0	precise
#043-6	EHA105	AMP-ORI	precise	12[b]	0	precise
#043-7	EHA105	AMP-ORI	precise	12[b]	0	precise
#043-8	EHA105	AMP-ORI	precise	12[b]	0	precise
#043-9	EHA105	AMP-ORI	precise	12[b]	0	precise
#043-10	EHA105	AMP-ORI	precise	12[b]	0	precise
#043-11	EHA105	AMP-ORI	precise	12[b]	0	precise
#043-12	EHA105	AMP-ORI	precise	12[b]	0	precise
#043-15	EHA105	AMP-ORI	precise	12[b]	0	precise
#043-16	EHA105	AMP-ORI	precise	12[b]	0	precise
#043-17	EHA105	AMP-ORI	precise	12[b]	0	precise
#043-18	EHA105	AMP-ORI	precise	12[b]	0	precise
#043-19	EHA105	AMP-ORI	precise	12[b]	0	precise
#043-20	EHA105	AMP-ORI	-294	1 (C)	0	-59
#043-21	EHA105	AMP-ORI	precise	12[b]	0	precise
#043-22	EHA105	AMP-ORI	precise	12[b]	0	precise
#043-23	EHA105	AMP-ORI	precise	12[b]	0	precise
#043-24	EHA105	AMP-ORI	precise	12[b]	0	precise
#043-26	EHA105	AMP-ORI	precise	12[b]	0	precise
#043-27	EHA105	AMP-ORI	precise	12[b]	0	precise
#043-28	EHA105	AMP-ORI	precise	12[b]	0	precise
#043-29	EHA105	AMP-ORI	precise	12[b]	0	precise
#043-31	EHA105	AMP-ORI	precise	12[b]	0	precise
#043-32	EHA105	AMP-ORI	precise	12[b]	0	precise
#043-33	EHA105	AMP-ORI	precise	12[b]	0	precise
#043-34	EHA105	AMP-ORI	precise	12[b]	0	precise
#043-35	EHA105	AMP-ORI	precise	12[b]	0	precise
#043-38	EHA105	AMP-ORI	precise	12[b]	0	precise
#043-39	EHA105	AMP-ORI	precise	12[b]	0	precise
#043-40	EHA105	AMP-ORI	precise	12[b]	0	precise
#043-43	EHA105	AMP-ORI	precise	12[b]	0	precise
#043-44	EHA105	AMP-ORI	precise	12[b]	0	precise
#043-45	EHA105	AMP-ORI	precise	12[b]	0	precise
#043-46	EHA105	AMP-ORI	precise	12[b]	0	precise
#043-47	EHA105	AMP-ORI	precise	12[b]	0	precise
#045-1	EHA105	AMP-ORI	precise	12[b]	0	precise
#045-2	EHA105	AMP-ORI	precise	12[b]	0	precise

#045-3	EHA105	AMP-ORI	precise	12[b]	0	precise
#045-4	EHA105	AMP-ORI	precise	12[b]	0	precise
#045-5	EHA105	AMP-ORI	precise	12[b]	0	precise
#045-6	EHA105	AMP-ORI	precise	12[b]	0	precise
#045-8	EHA105	AMP-ORI	precise	12[b]	0	precise
#045-9	EHA105	AMP-ORI	precise	12[b]	0	precise
#045-10	EHA105	AMP-ORI	precise	12[b]	0	precise
#045-11	EHA105	AMP-ORI	precise	12[b]	0	precise
#045-14	EHA105	AMP-ORI	precise	12[b]	0	precise
#045-15	EHA105	AMP-ORI	precise	12[b]	0	precise
#045-16	EHA105	AMP-ORI	precise	12[b]	0	precise
#045-17	EHA105	AMP-ORI	precise	12[b]	0	precise
#045-18	EHA105	AMP-ORI	precise	12[b]	0	precise
#045-20	EHA105	AMP-ORI	precise	12[b]	0	precise
#045-21	EHA105	AMP-ORI	precise	12[b]	0	precise
#045-22	EHA105	AMP-ORI	precise	12[b]	0	precise
#045-24	EHA105	AMP-ORI	precise	12[b]	0	precise
#045-25	EHA105	AMP-ORI	precise	12[b]	0	precise
#045-27	EHA105	AMP-ORI	precise	12[b]	0	precise
#045-28	EHA105	AMP-ORI	precise	12[b]	0	precise
#045-29	EHA105	AMP-ORI	precise	12[b]	0	precise
#045-30	EHA105	AMP-ORI	precise	12[b]	0	precise

[a]Right border (RB) and left border (LB) numerical values represent the position in DNA relative to a precise end.

[b]Microhomology of 12 bp nucleotides is true only of there were a readthrough of a RB sequence. Alternately, precise RBs and precise LBs were joined without microhomology.

Supplemental Table 7. Bacterial strains used in this study

Strains & plasmids	Description/Use	Antibiotic resistance[1]	Reference
***E. coli*:**			
DH10B	F– *mcrA* Δ(*mrr-hsdRMS-mcrBC*) φ80*lacZ*ΔM15 Δ*lacX74 recA1 endA1 araD139* Δ (*ara-leu*)7697 *galU galK* λ– *rpsL*(Str^R) *nupG*	None	Grant et al., 1990
E4	*E. coli* 2104 containing pPH1JI	Gent	Hirsch and Beringer, 1984
E1500	pUC18 with P_{virD}-*VirD1*-*VirD2* ω mutation	Amp	Gelvin lab stock
E1727	pLAFR1 containing an *Eco*RI fragment containing a modified *picA* locus	Tet	Gelvin lab stock
E1745	5.5 kbp blunted *Eco*RI fragment of pE1500 cloned to the blunted *Pst*I site of pE1727	Amp, Tet	Gelvin lab stock
E1961	2.6 kbp *Pst*I fragment containing the *sacRB* genes cloned into the *Pst*I site of pBluescript KS-	Amp	Gelvin lab stock
E3052	*Eco*RI-*Sac*II fragment containing the *VirD2* gene cloned into the corresponding sites of pSAT6-nEYFP-C1	Amp	Gelvin lab stock
E3332	7.2 kbp *Xho*I fragment containing the *VirD* operon cloned into the *Xho*I site of pBluescript KS+	Amp	Gelvin lab stock
E3351	Klenow filled-in *Asp*718 site of pBluescript KS+	Amp	Gelvin lab stock
E3353	3.27 kbp blunted *Sph*I-XhoI fragment of pE3332 cloned into the *Sma*I-*Xho*I sites of pE3351	Amp	Gelvin lab stock
E3355	*Hin*dIII fragment from pE3052 cloned into the *Hin*dIII site of pE3353	Amp	Gelvin lab stock
E3356	*Kpn*I deletion of pE3355 to create a non-polar deletion of *VirD2*	Amp	Gelvin lab stock
E3358	*Xho*I-*Not*I fragment from pE3356 cloned into the corresponding sites of pJQ200sk	Gent	Gelvin lab stock
E4329	pPZP-*hpt*-Venus-intron binary vector	Spec	Gelvin lab stock
E4579	pE4330 ligated to pUC19 at the *Sal*I and *Sac*I sites	Amp, Spec	This study
E4636	T-circle binary vector	Spec	This study
E4252	pRCS11 (TET-ORI; KS101)	Tet, Spec	This study
E4253	pTET-ORI modified RB region; KS102	Tet, Spec	This study
E4254	TT3369; pAMP-ORI T-circle binary vector	Amp, Spec	Singer et al., 2012
E4255	TT4500; pKAN-ORI T-circle binary vector	Kan, Spec	Singer et al., 2012
pEHC13	Cosmid clone of pTiBo542 Vir region		Hood et al., 1984
pJQ200sk	*sacRB* plasmid	Gent	Quandt and Hines, 1993
A. tumefaciens:			
A136	Strain C58 cured of the Ti-plasmid	Rif	Watson et al., 1975
EHA105	Disarmed super-virulent strain	Rif	Hood et al., 1993
At1132	pE1745 in A136	Carb, Rif, Tet	Gelvin lab stock
At1136	Transconjugant from At1132 x E4	Carb, Rif	Gelvin lab stock
At1697	EHA105 with a non-polar *VirD2* deletion	Rif	This study
At1710	pTiBo542-ΔVirD2 from At1697 with the VirD2 ω substitution in At1136 + pPH1JI	Carb, Gent, Rif, Spec	Gelvin lab stock

At1959	pTiBo542-ΔVirD2 from At1697 with the VirD2 ω substitution lacking pPH1JI	Carb, Rif	This study
At2120	EHA105(pBISN1, pKS102)	Kan, Rif, Spec	This study
At2121	At1959(pBISN1, pKS102)	Kan, Rif, Spec	This study
At2162	At1959(pBISN1, pRCS11)	Carb, Kan, Rif, Spec	This study
At2168	pRCS11 in At1959	Carb, Spec	This study
At2273	pE4636 T-circle binary vector in EHA105	Carb, Rif, Spec	This study
At2332	pE4636 T-circle binary vector in A1959	Carb, Rif, Spec	This study

[1]Amp, ampicillin; Carb, carbenicillin; Gent, gentamicin; Kan, kanamycin; Rif, rifampicin; Spec, spectinomycin; Tet, tetracycline
