# Supplementary material for: Characterization of T-Circles and Their Formation Reveal Similarities to Agrobacterium T-DNA Integration Patterns
Source: Front Plant Sci. 2022 May 6;13:849930. doi: 10.3389/fpls.2022.849930 (PMC9121065; doi:10.3389/fpls.2022.849930)

Supplemental Figure 1—Characterization of complex T-circles from *Nicotiana benthamiana*

T-circles which were re-classified as complex after DNA sequence analysis of T-DNA junctions.

Blue: T-DNA sequence; Green: Non-T-DNA sequence (binary vector, Ti plasmid, or unknown (“scrambled”) filler DNA.

#002-23

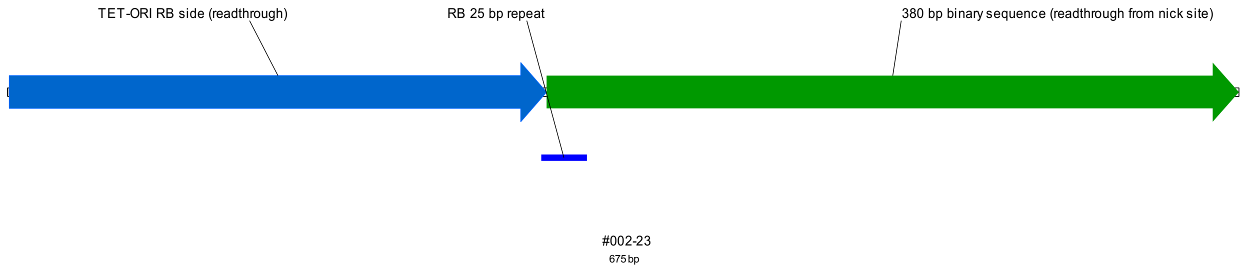

#003-50

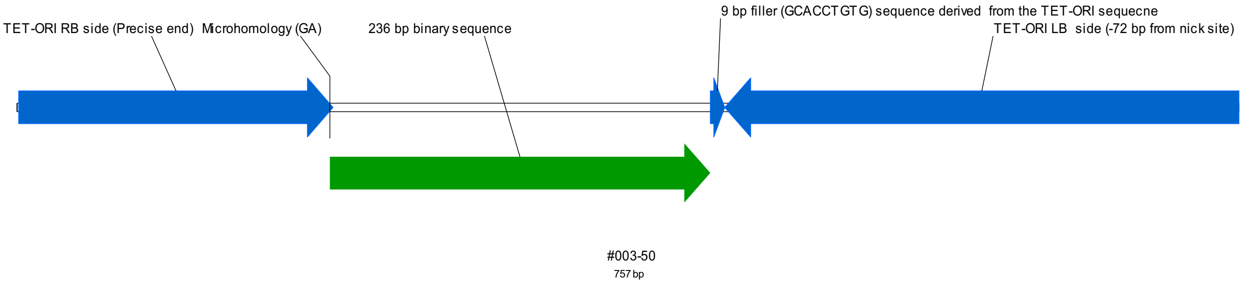

#003-61

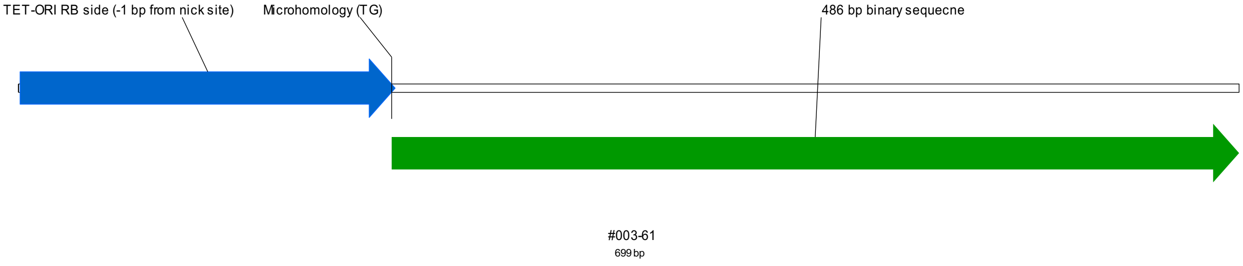

#003-62

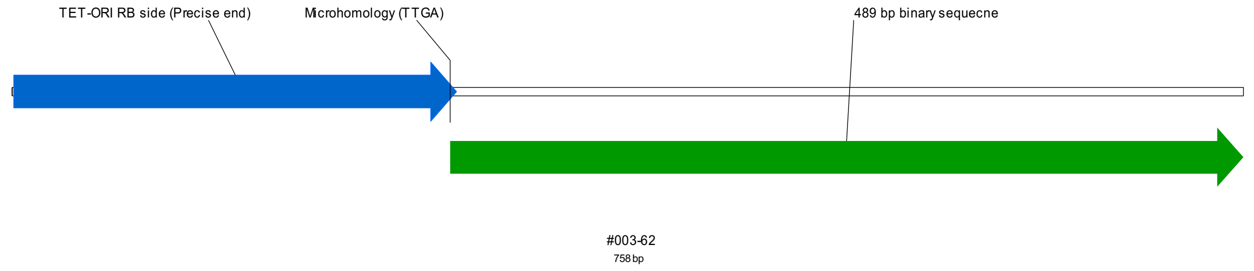

#003-64

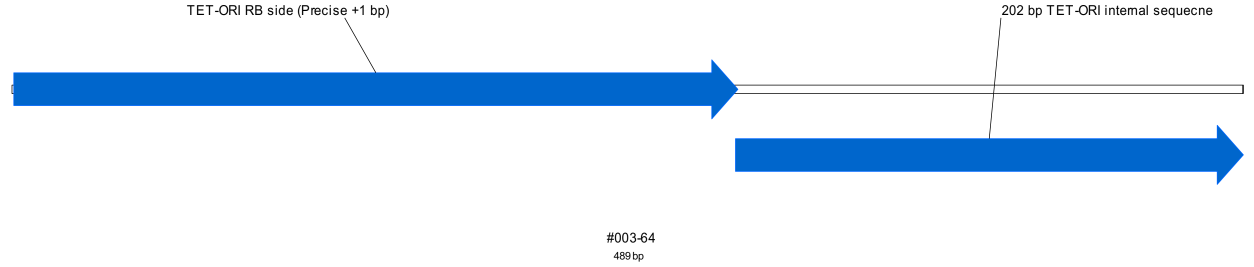

#005-8

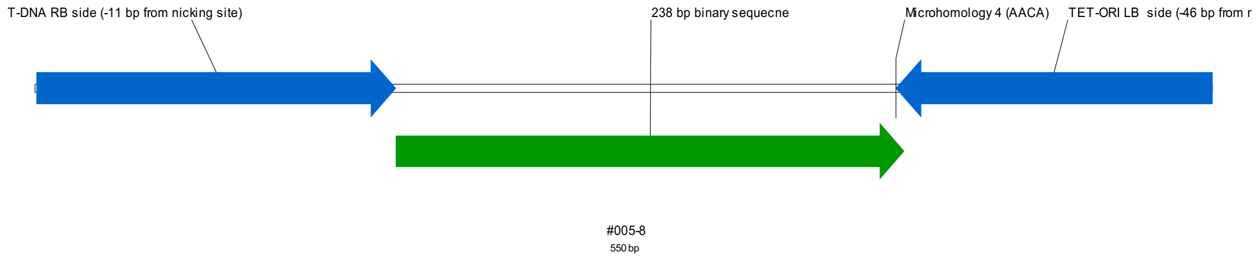

#008-73

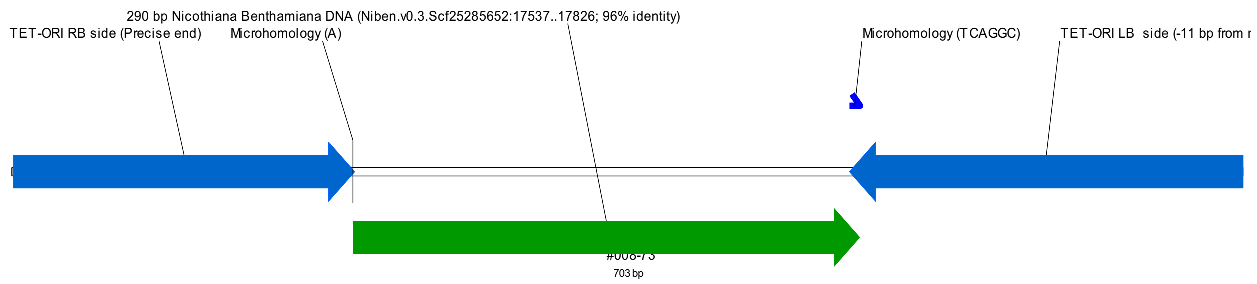

#009-12

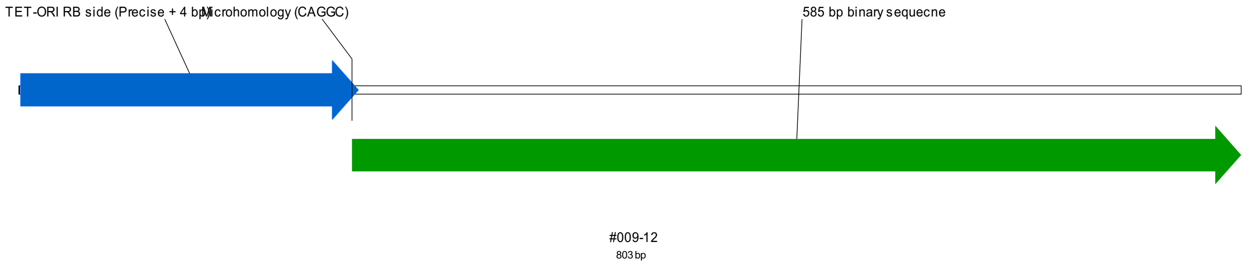

#050-14

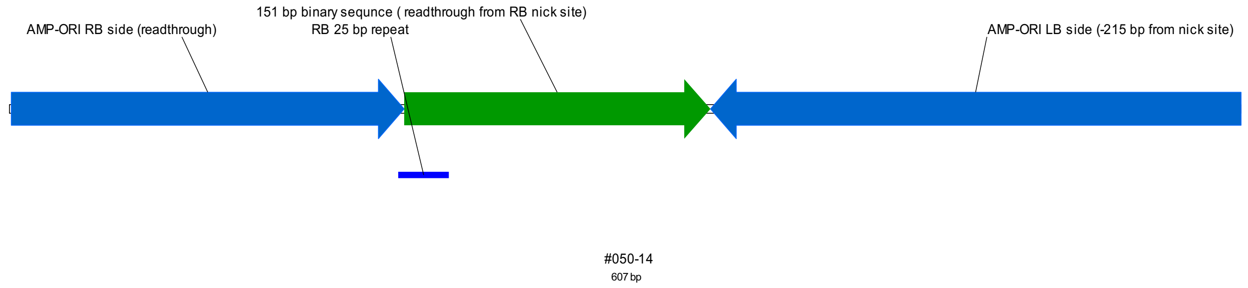

#050-23

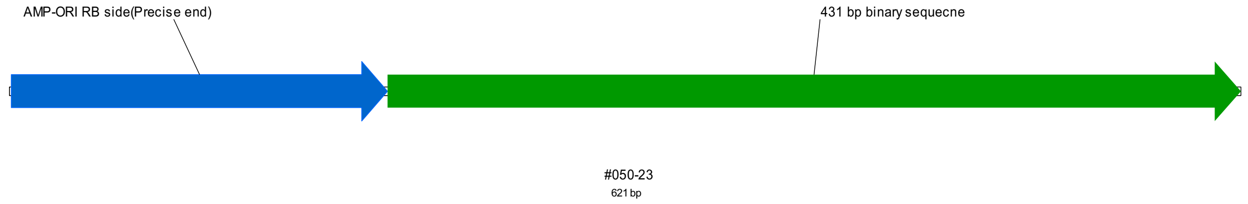

#052-9

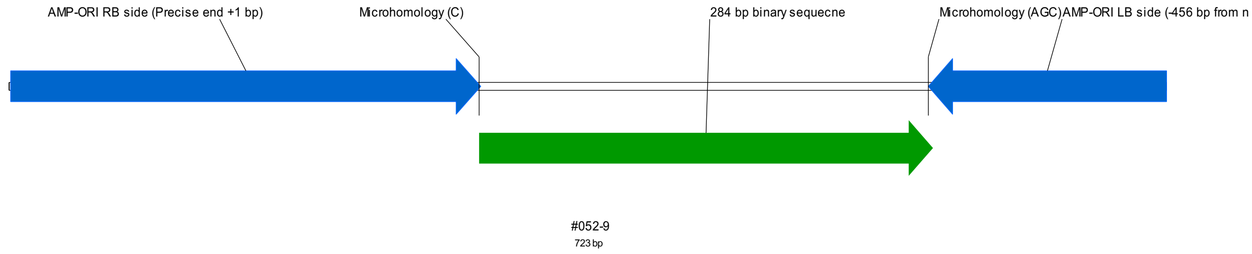

#052-17

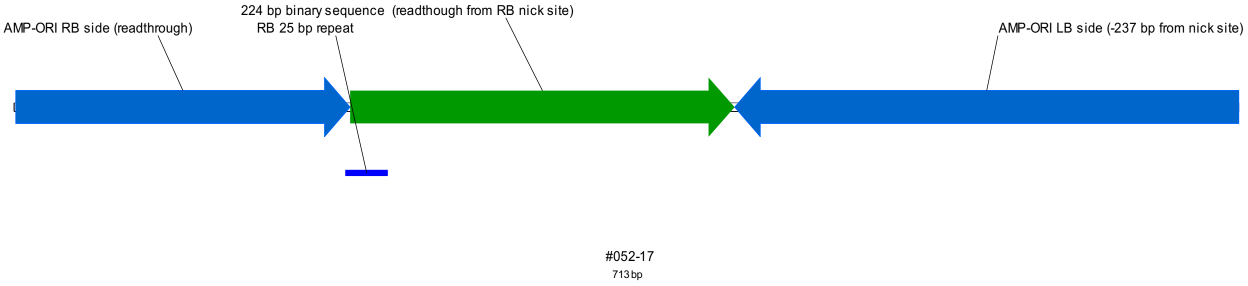

#052-18

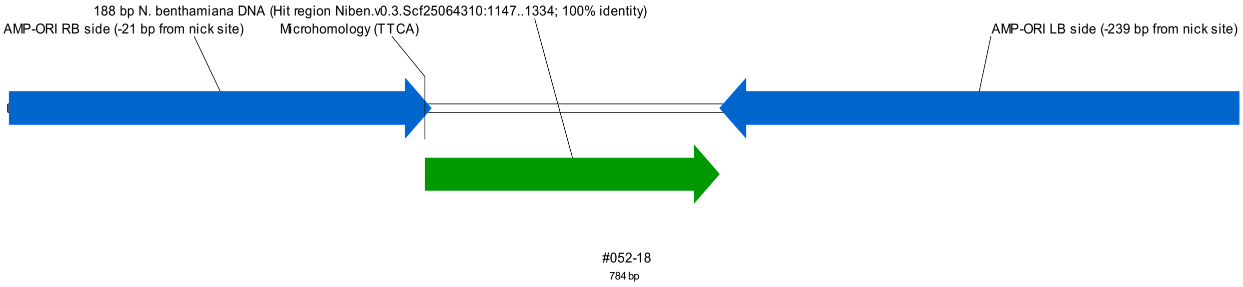

#052-22

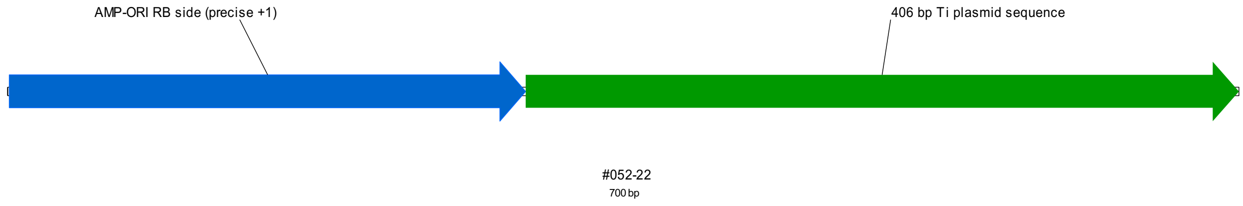

#052-28

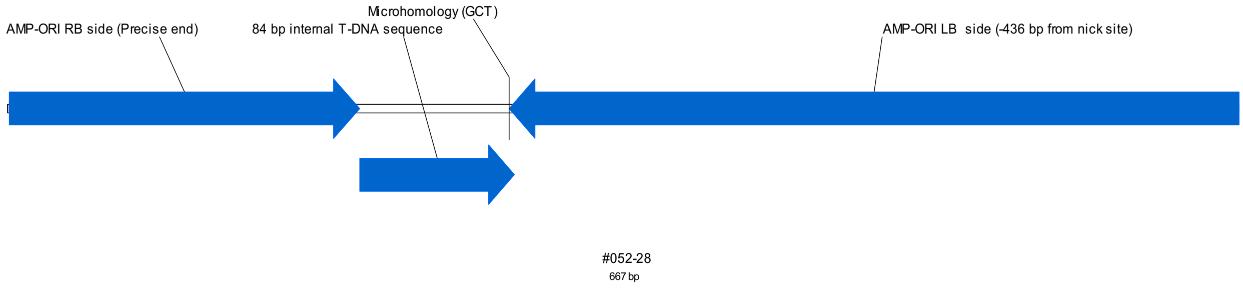

#052-35

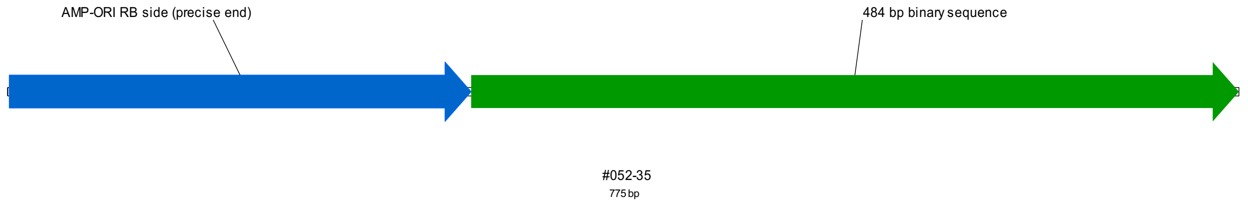

#052-44

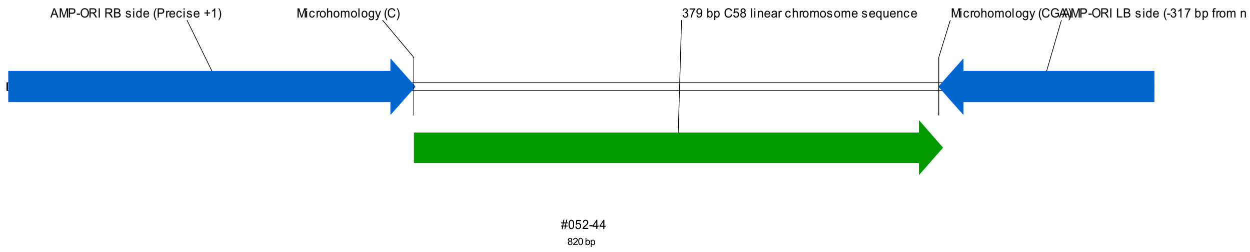

#052-45

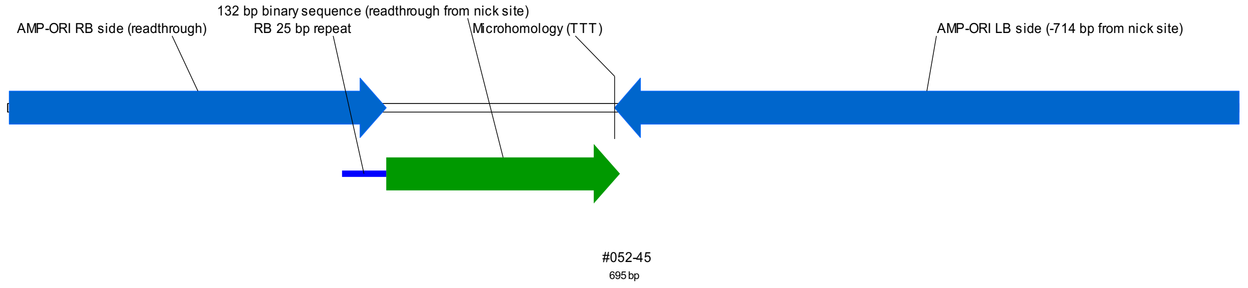

#055-55

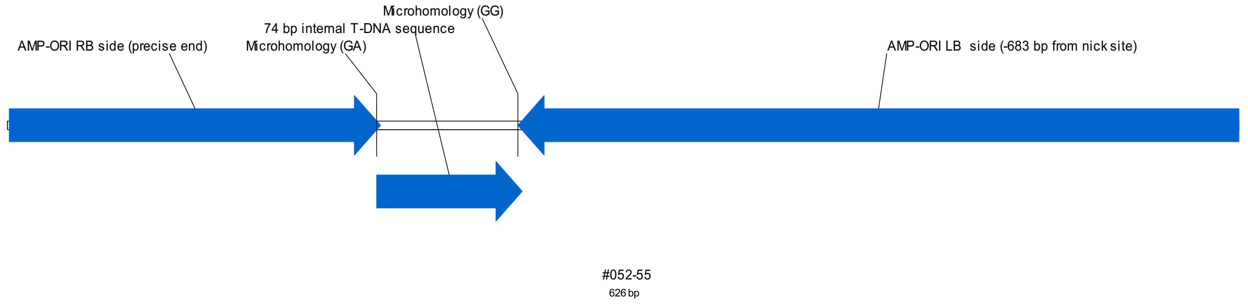

#052-60

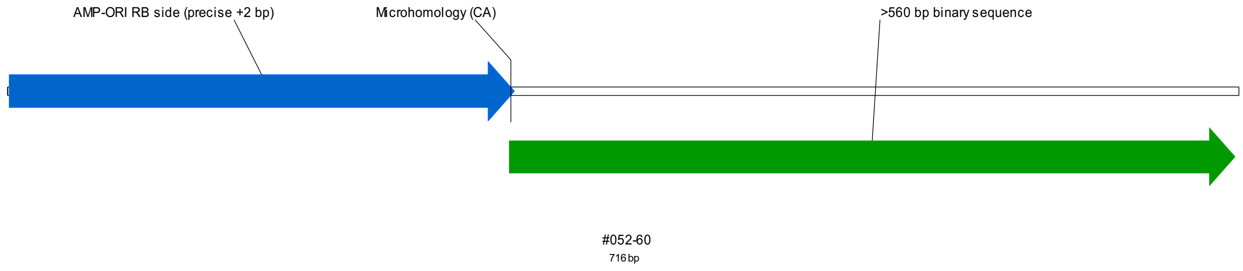

#052-63

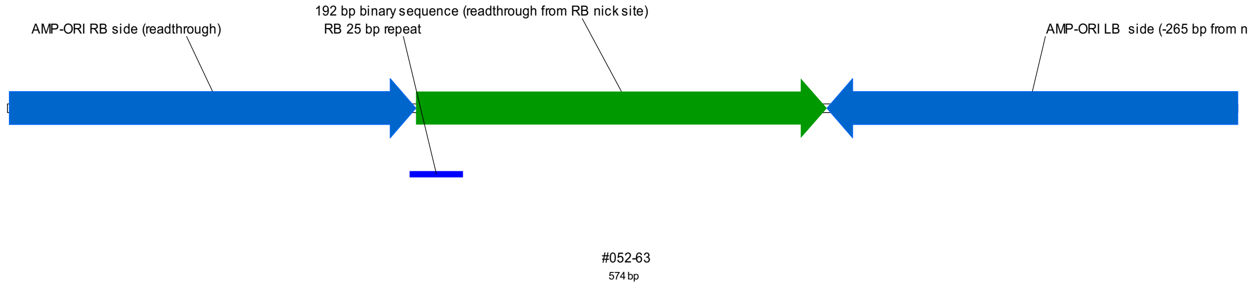

#052-66

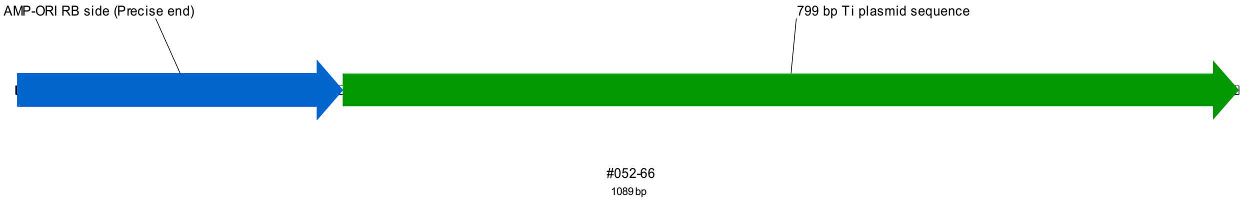

#021-4

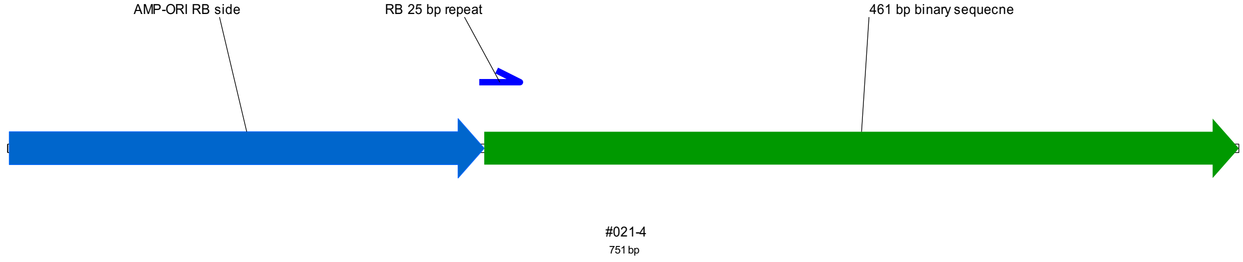

Supplemental Figure 2

Schematic representation of the complex T-circle #12. RB and LB, T-DNA right and left borders, respectively. tetR and KanR, genes encoding tetracycline and kanamycin resistance, respectively.

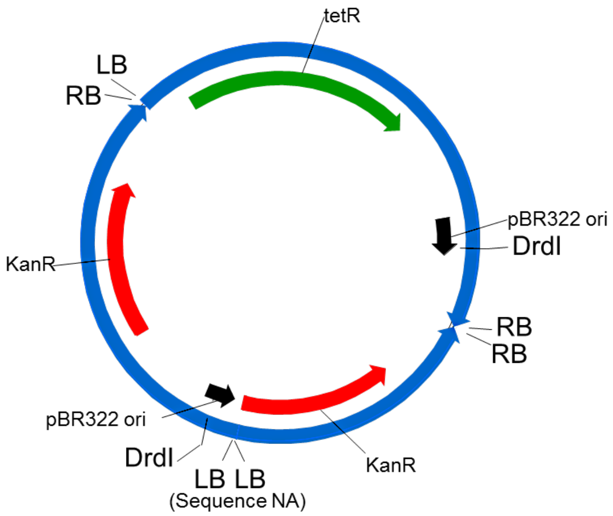

Supplemental Figure 3—Characterization of heterodimeric T-circles

T-circle junctions made up of two different T-DNAs, based on DNA sequence analysis.

#3 LB-LB:

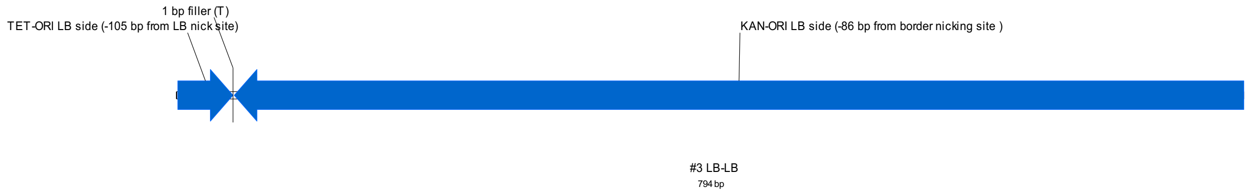

#3 RB-RB

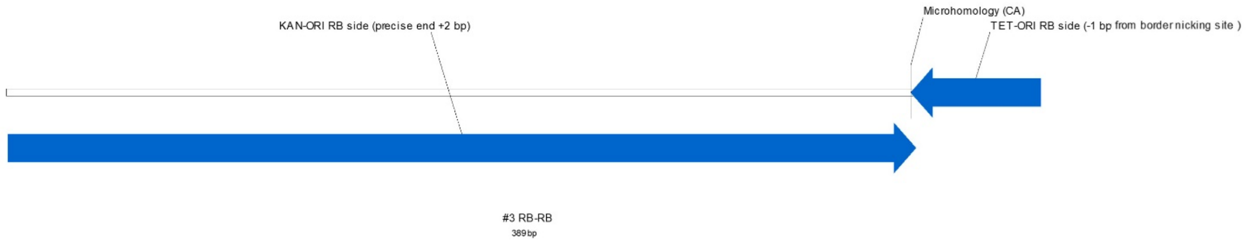

#4 LB-LB:

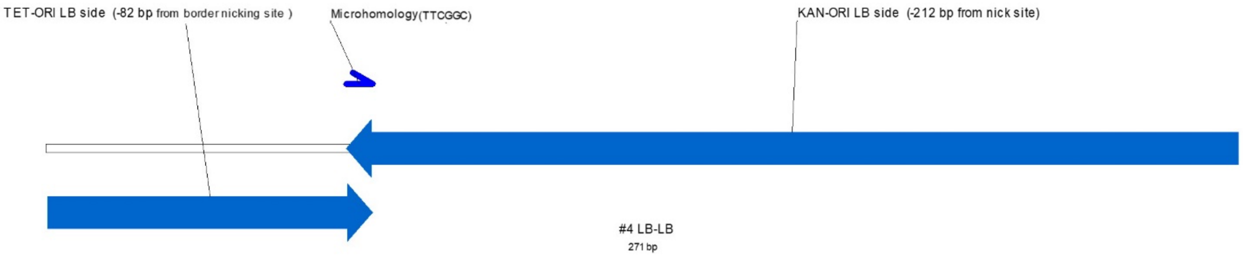

#4 RB-RB

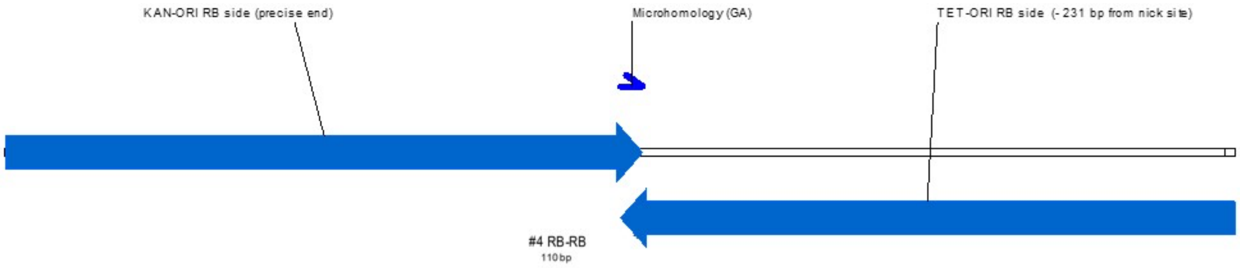

#5 LB-LB

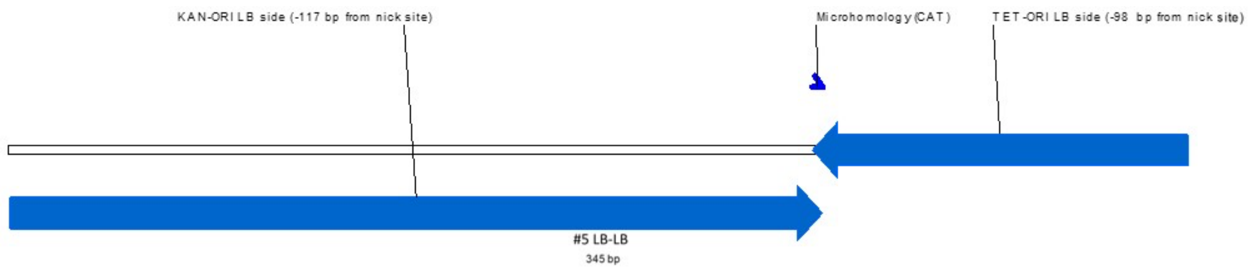

#5 RB-RB

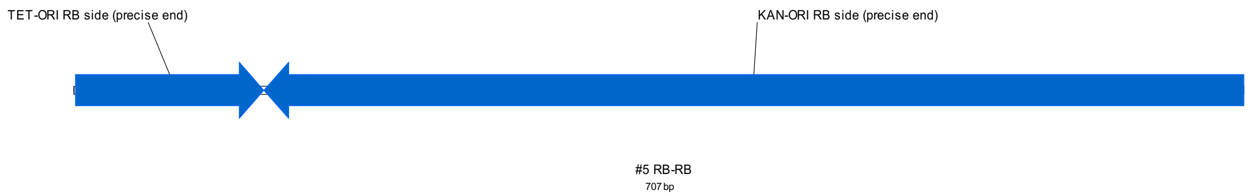

#6 LB-LB

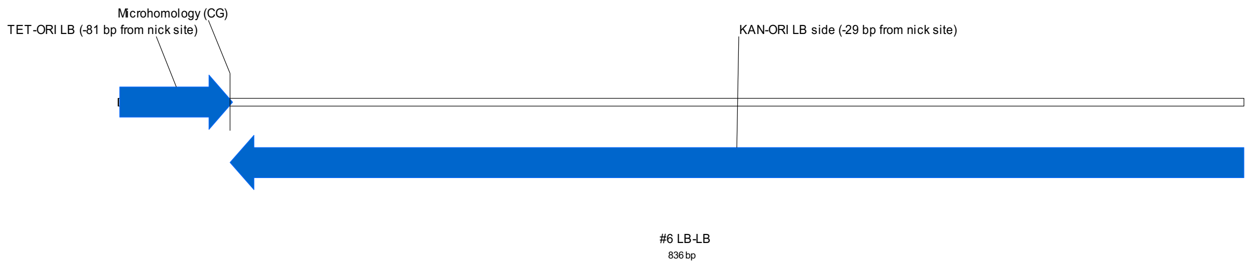

#6 RB-RB

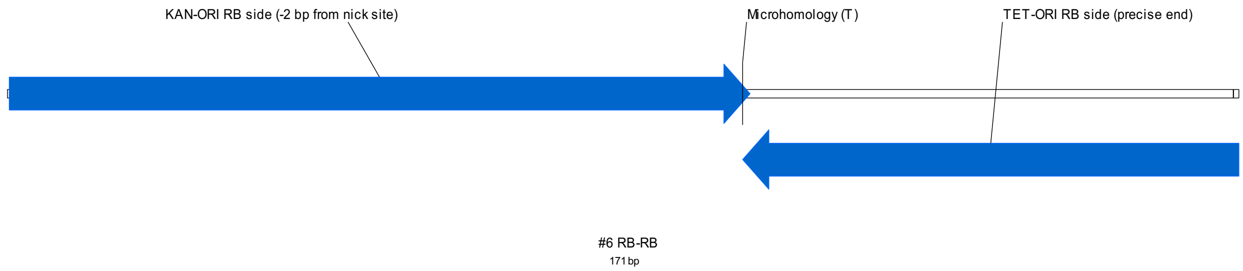

#9 LB-LB:

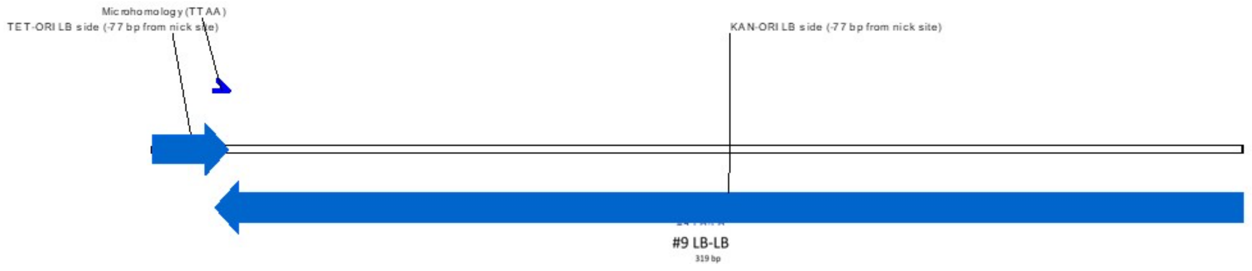

#9 RB-RB:

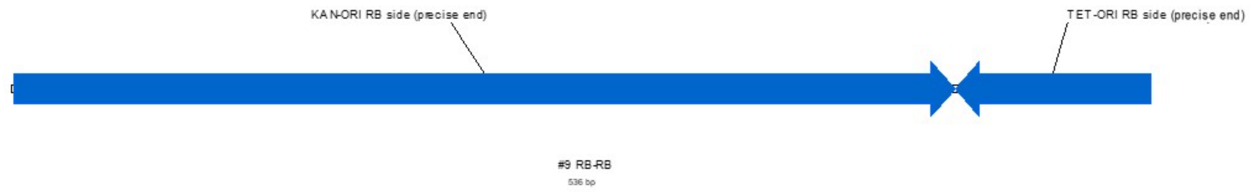

#10 LB-LB:

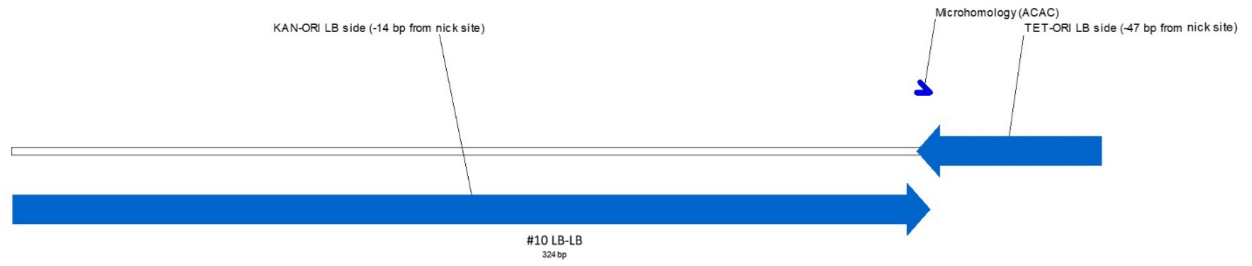

#10 RB-RB:

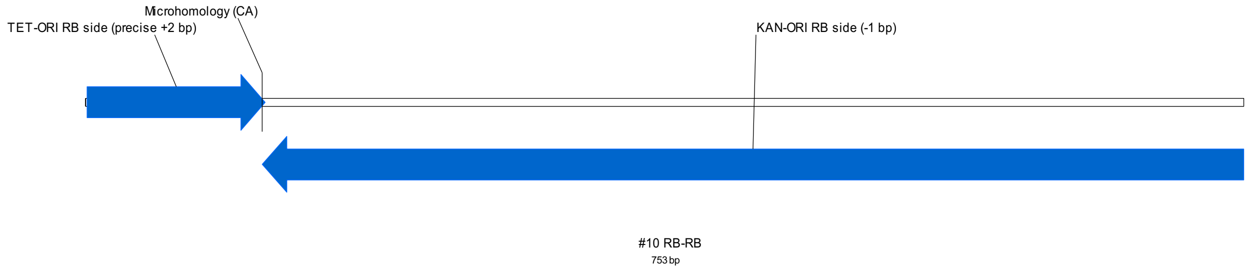

#11 LB-LB:

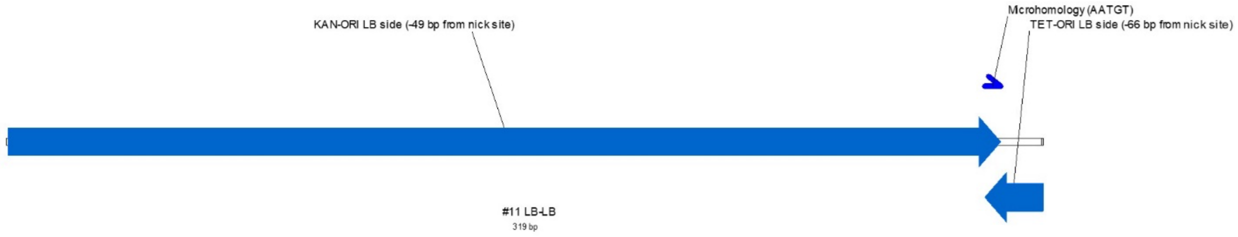

#11 RB-RB:

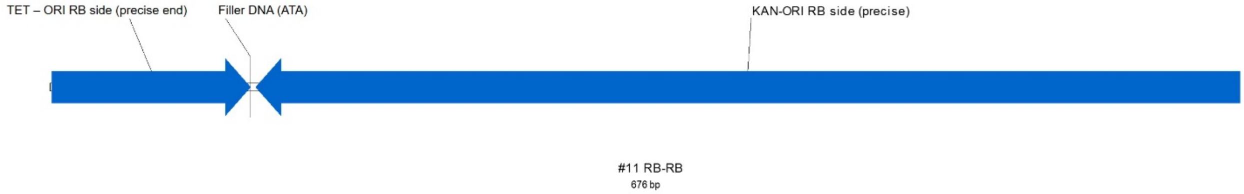

#12 LB-RB:

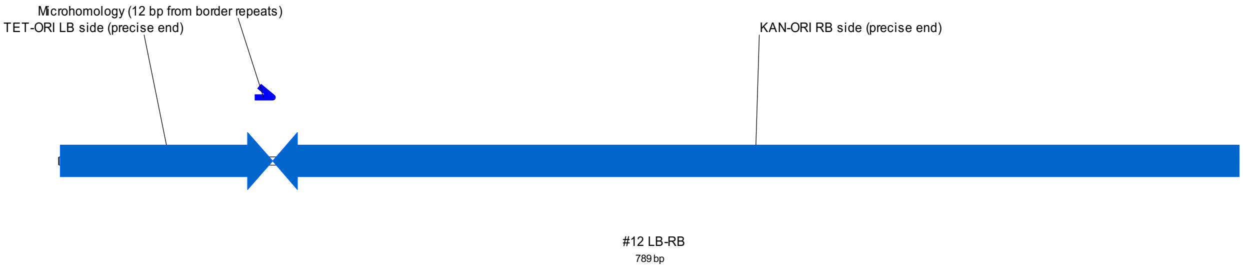

#12 RB-RB:

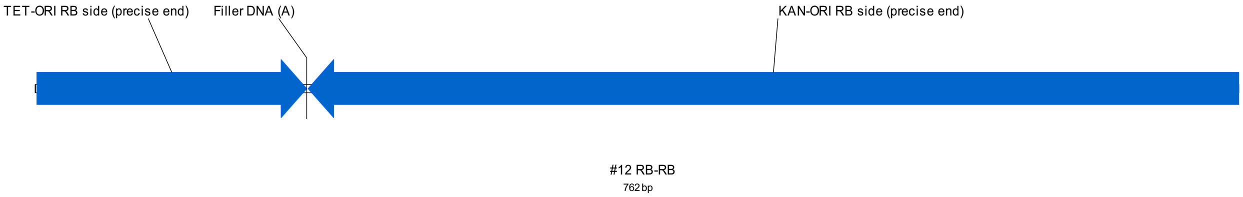

#15 LB-LB:

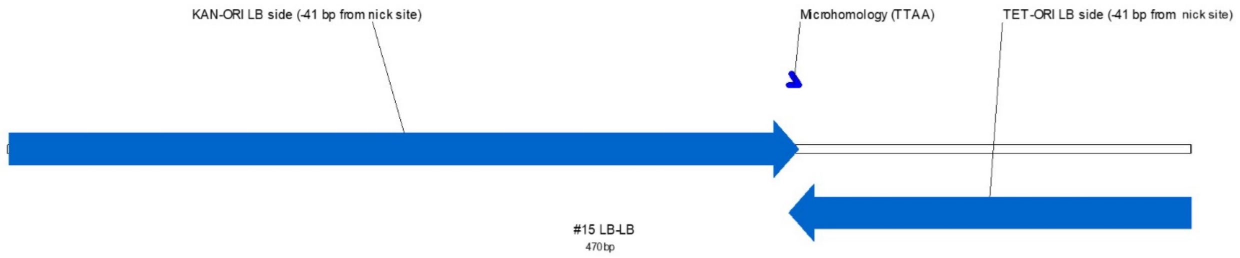

#15 RB-RB:

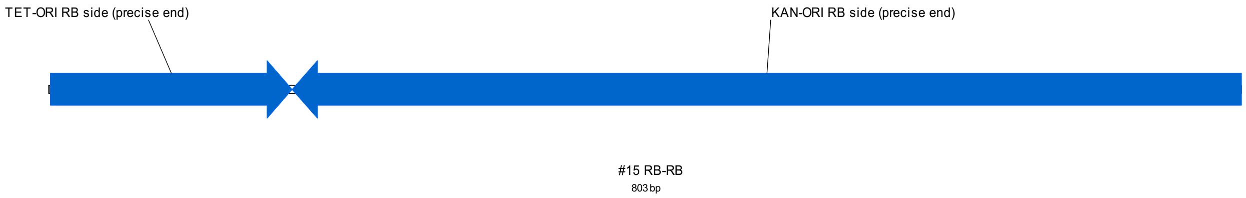

#16 LB-LB:

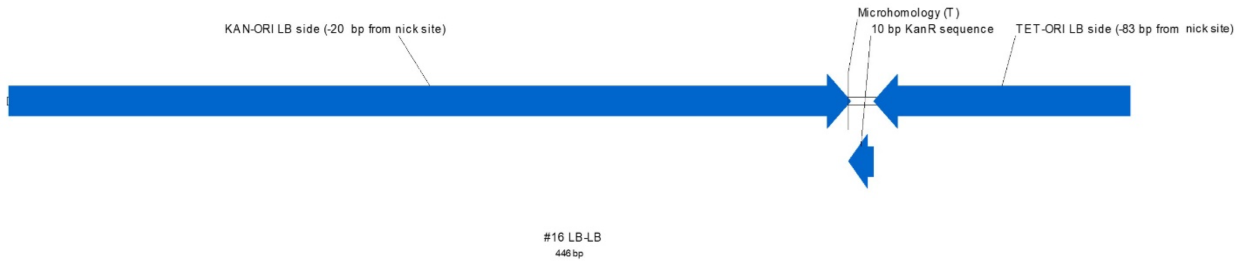

#16 RB-RB:

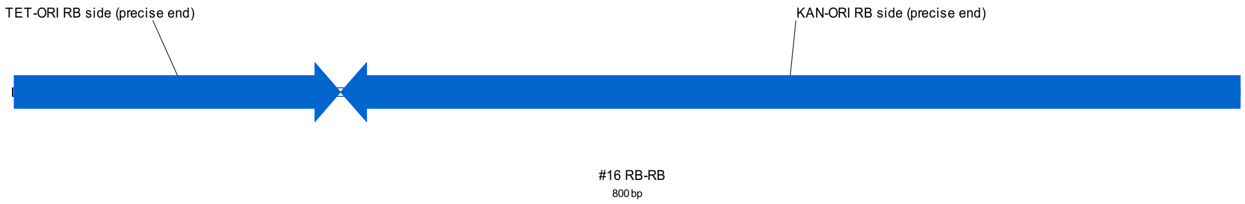

#17 LB-RB:

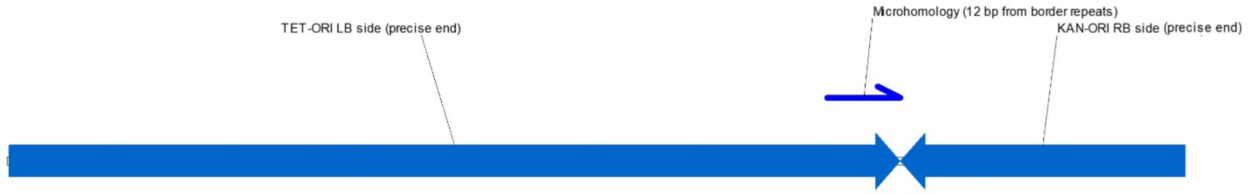

#17 LB(TET-ORI)-RB(KAN-ORI)
194 bp

#17 LB-RB:

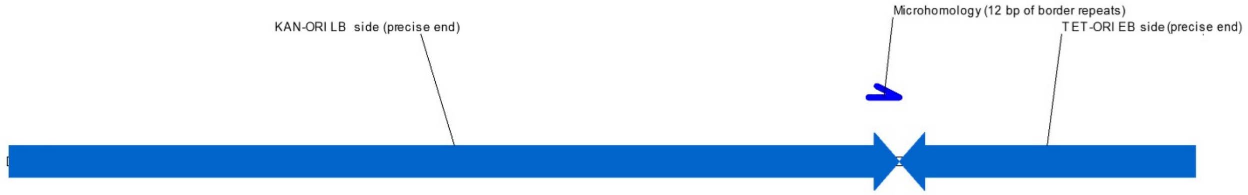

#17 LB(KAN-ORI)-RB(TET-ORI)
461 bp

#18 LB-LB (complex structure):

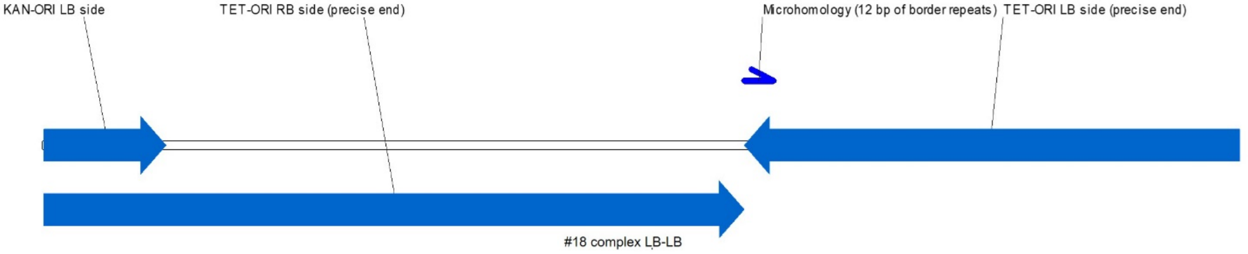

#18 complex LB-LB
490 bp

#18 RB-RB:

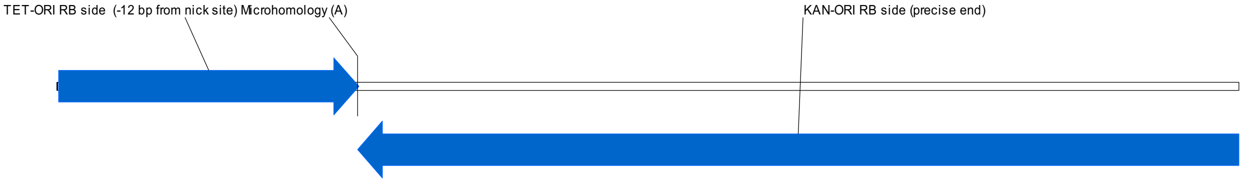

#18 RB-RB
861 bp

#22 LB-LB:

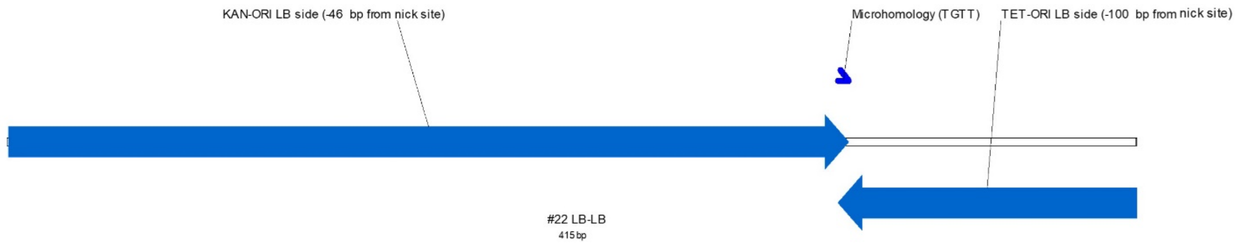

#22 RB-RB

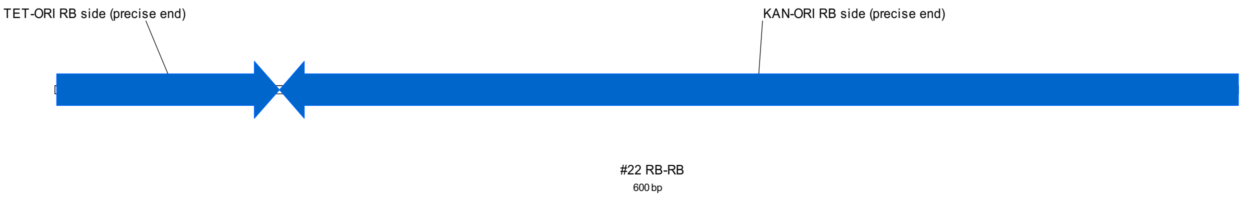

#24 LB-LB:

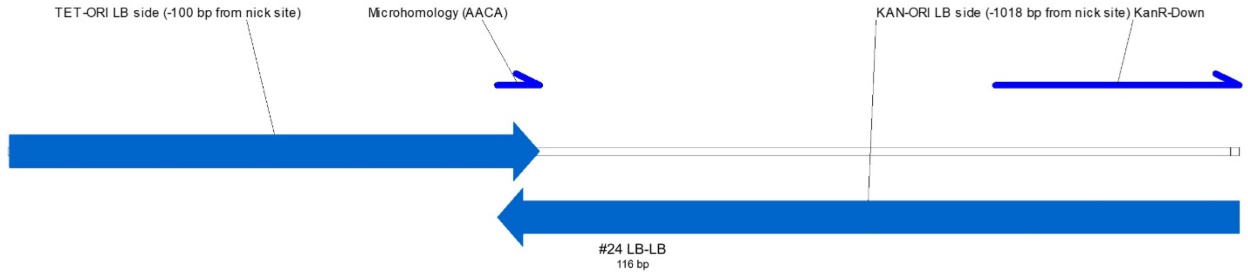

#24 RB-RB:

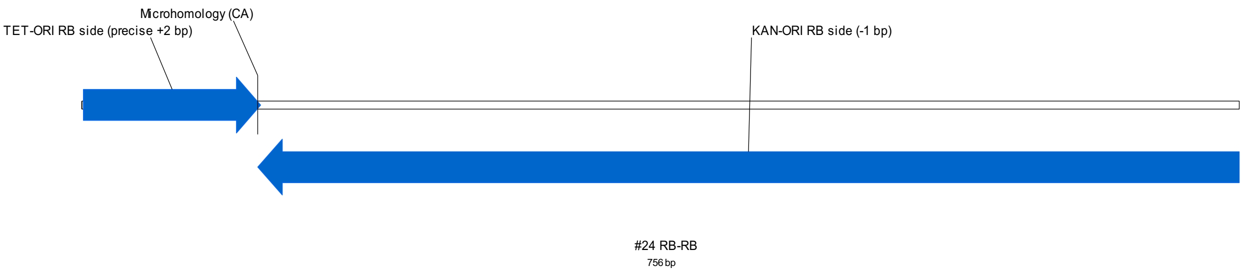

#25 LB-LB

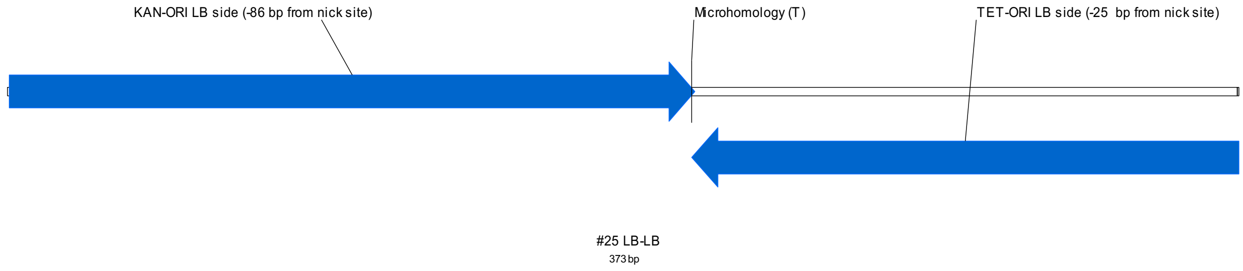

#25 RB-RB

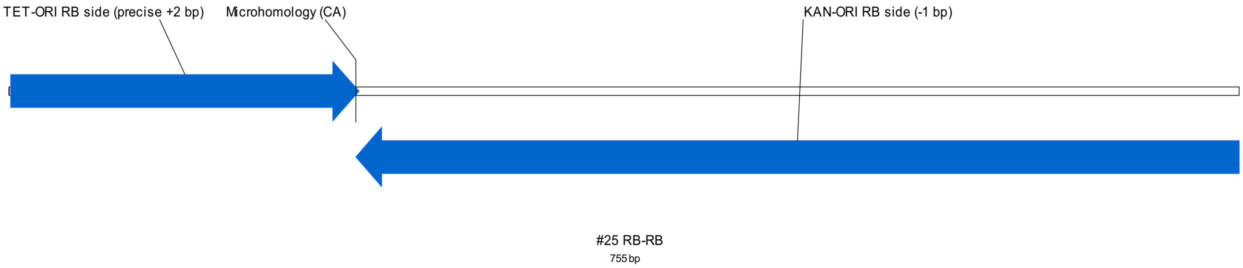

#30 RB-RB:

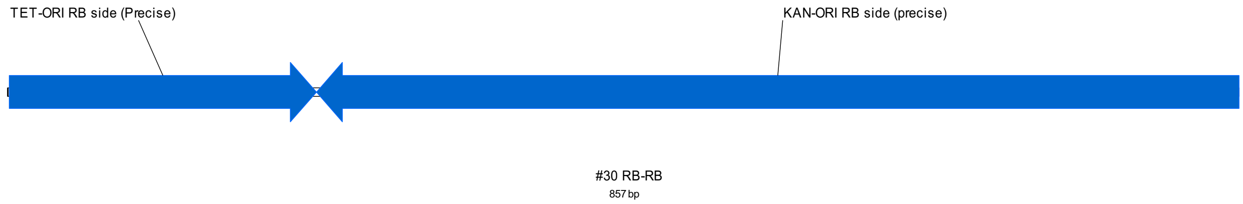

#32 LB-LB:

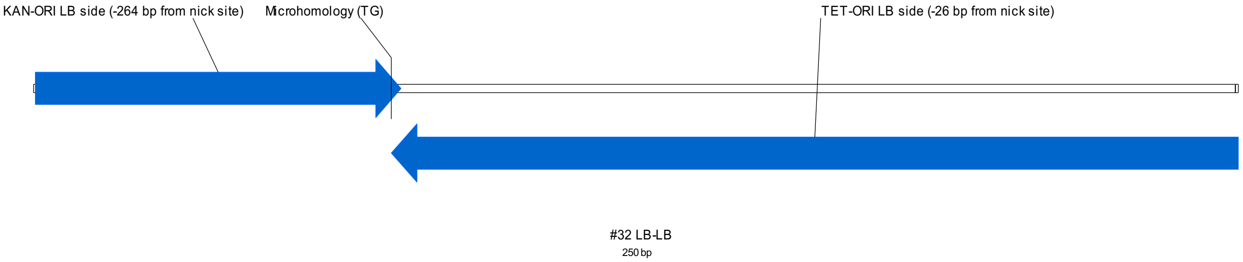

#32 RB-RB:

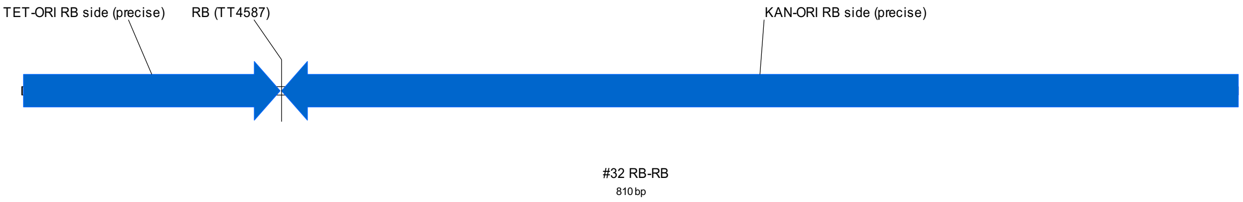

#37 LB-LB

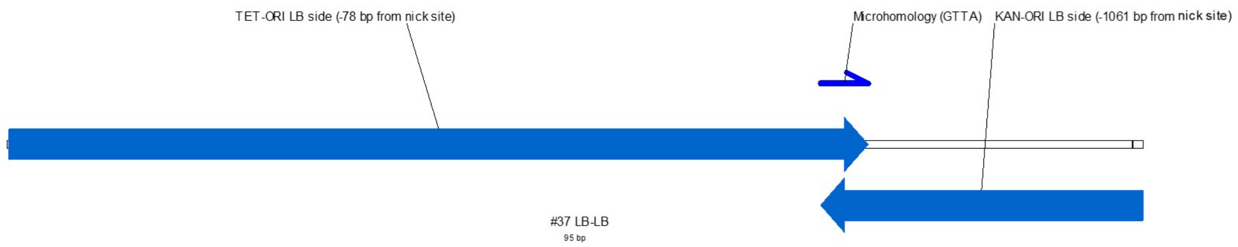

#37 RB-RB:

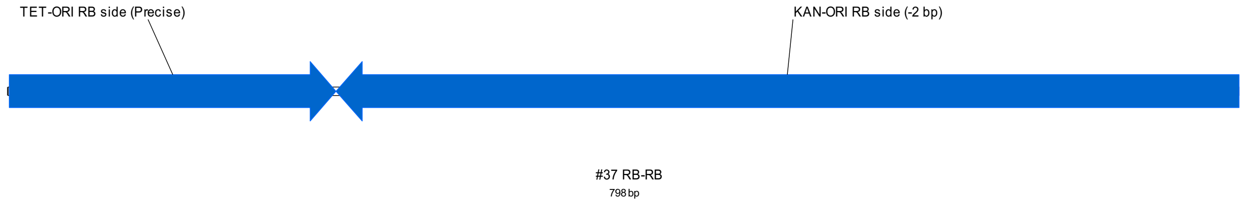

Supplement: Supplementary file 2 [file Data_Sheet_2.PDF]
